# Morphological Characterization and Integrated Transcriptome and Proteome Analysis of *Organ Development Defective 1* (*odd1*) Mutant in *Cucumis sativus* L.

**DOI:** 10.3390/ijms23105843

**Published:** 2022-05-23

**Authors:** Jing Han, Zengguang Ma, Linjie Chen, Zaizhan Wang, Can Wang, Lina Wang, Chunhua Chen, Zhonghai Ren, Chenxing Cao

**Affiliations:** State Key Laboratory of Crop Biology, Shandong Collaborative Innovation Center of Fruit & Vegetable Quality and Efficient Production, Key Laboratory of Biology and Genetic Improvement of Horticultural Crops in Huang-Huai Region, Ministry of Agriculture, College of Horticultural Science and Engineering, Shandong Agricultural University, Tai’an 271018, China; jhan@sdau.edu.cn (J.H.); 2020120409@sdau.edu.cn (Z.M.); 2020110315@sdau.edu.cn (L.C.); 2021120444@sdau.edu.cn (Z.W.); 2019110225@sdau.edu.cn (C.W.); lnwang@sdau.edu.cn (L.W.); chenchunhua@sdau.edu.cn (C.C.)

**Keywords:** cucumber, organ development, *odd1* mutant, transcriptomics, proteomics

## Abstract

Cucumber (*Cucumis sativus* L.) is an economically important vegetable crop with the unique growth habit and typical trailing shoot architecture of Cucurbitaceae. Elucidating the regulatory mechanisms of growth and development is significant for improving quality and productivity in cucumber. Here we isolated a spontaneous cucumber mutant *organ development defective 1* (*odd1*) with multiple morphological changes including root, plant stature, stem, leaf, male and female flowers, as well as fruit. Anatomical and cytological analyses demonstrated that both cell size and number decreased, and the shoot apical meristem (SAM) was smaller in *odd1* compared with WT. Pollen vigor and germination assays and cross tests revealed that *odd1* is female sterile, which may be caused by the absence of ovules. Genetic analysis showed that *odd1* is a recessive single gene mutant. Using the MutMap strategy, the *odd1* gene was found to be located on chromosome 5. Integrated profiling of transcriptome and proteome indicated that the different expression genes related to hormones and SAM maintenance might be the reason for the phenotypic changes of *odd1*. These results expanded the insight into the molecular regulation of organ growth and development and provided a comprehensive reference map for further studies in cucumber.

## 1. Introduction

Cucumber (*Cucumis sativus* L.) is an economically important vegetable crop worldwide, which has the unique growth habit and shoot architecture of Cucurbitaceae [1,2,3,4]. The vegetative and reproductive growth of cucumber proceed simultaneously after a very short juvenile stage [5,6,7]. Leaves are derived from the periphery of the shoot apical meristem (SAM). Then unisexual (male or female) flowers and tendrils occur in the leaf axils [8]. Finally, a typical vining cucumber plant formed. The generation of cucumber architecture requires a fine and coordinated process [3]. The ability to regulate shoot architecture is very useful to improve planting density and productivity and cultivation efficiency in cucumber breeding. However, the understanding of the mechanisms controlling shoot architecture in cucumber is still limited. Therefore, elucidating the regulatory molecular mechanisms of cucumber growth and development is necessary.

Developmental mutants have played important roles in the study of cucumber growth and development. Several types of developmental mutants have been described in cucumber. They are mainly divided into dwarf mutants, leaf color and leaf shape mutants, flower organ development mutants, and fruit development mutants, for example, dwarf or compact mutants with reduced vine length including *compact* (*cp*) [9], *compact-2* (*cp-2*) [10], and *super compact* (*scp*) [11]. As the candidate gene of *super compact-1* (*scp-1*), *CsCYP85A1* encoding the brassinosteroid (BR)-6-oxidase in the BR biosynthesis pathway has been reported as the first cloned gene for plant height in cucurbit crops through map-based cloning [11]. Furthermore, *CsDET2* was cloned as the candidate gene for a BR biosynthesis-deficient dwarf mutant *super compact-2* (*scp-2*) [12]. Mutants with abnormal leaf morphology include *round leaf* (*rl-1*) mutant [13], *little leaf* (*ll*) mutant [14], and *mango fruit* (*mf*) mutant [15]. Through the genetic analysis of the *mf rl* double mutant, CsWOX1 regulates leaf vein patterning by CsPID-mediated auxin transport. Moreover, CsWOX1 regulates leaf development by interacting with CIN (CINCINNATA)-TCP (TEOSINTE BRANCHED1/CYCLOIDEA/PCF) proteins [15]. Using map-based cloning, the determinate growth habit gene *CsTFL1* was isolated. CsTFL1 inhibits determinate growth and terminal flower formation by interacting with CsNOT2a [16]. The *short fruit 1* (*sf1*) mutant bears short fruits owing to repressed cell division. The *SF1* gene encodes a cucurbit-specific RING-type E3 ligase, which ubiquitinates and degrades both itself and ACS2 (1-aminocyclopropane-1-carboxylate synthase 2) to control ethylene synthesis for a dose-dependent effect on cell division and fruit elongation in cucumber [17]. Moreover, many natural (or artificial) mutants were also used to analyze organ or tissue development in cucumber [18,19,20,21]. In addition, some of the genes related to growth and development were identified by means of reverse genetics in cucumber. There have been about 25 genes reported as being involved in shoot architecture of cucumber, including determinate growth habit (*CsTFL1* and *CsLFY*), leaf morphology (*CsPID*, *CsWOX1*, *CsIVP*, *CsYAB5*, *CsPHB*, *CsSAP*, *CsSL1*, and *CsHAN1*), lateral branching (*CLS*, *CsBRC1*, and *CsPIN3*), tendril formation (*CsTEN*, *CsACO1*, *CsGCN5*, and *CsPID*), and vine length (*CsSH1*, *CsGPA1*, *CsCLV1*, *CsCKX*, *CsCullin1*, *CsCYP85A1*, *CsDET2*, *CsVFB1*, *CsIVP*, and *CsYAB5*) [3]. Although a large number of developmental mutants has been reported and some candidate genes related to plant development cloned, the molecular mechanisms underlying plant growth and development in cucumber remains unclear.

Genes underlying growth and development in a number of plant species have been cloned and functionally characterized. In Arabidopsis, the CLAVATA (CLV)-WUSCHEL (WUS) negative feedback loop is a major genetic mechanism to maintain meristem homeostasis [22]. *WUS* is essential for meristem function because the stem cells are mis-specified and appear to undergo differentiation in the *Arabidopsis wus* mutant [23]. Mutations in the *CLV* genes, such as *CLV1*, *2*, and *3*, cause the enlargement of the meristem [24]. *WUS* is expressed in the organizing center (OC) cells, and the WUS protein moves from the OC to the central zone (CZ) and promotes *CLV3* expression. CLV3 in turn moves to the OC cells to restrict the transcription of *WUS* [25]. The negative feedback regulation between WUS and CLV3 affects the activity of the stem cell microenvironment and determines the generation of the organizing center and stem cells [26,27,28,29]. Moreover, the expression of *WUS* and *CLV3* is activated by cytokinin (CK), whereas it is repressed by auxin [30]. WUS is one of the WUSCHEL RELATED HOMEOBOX (WOX) proteins, which belong to the plant HB (homeobox transcription factors) family, Knotted Related Homeobox (KNOX), and play positive roles in key developmental processes in plants, such as embryonic patterning, stem-cell maintenance, and organ formation by promoting cell division activity and preventing premature cell differentiation [31]. For example, *WOX4*, a member of the *WOX* gene family, acts as a positive factor in shoot meristem maintenance and is repressed by *FCP1* in rice [32]. Plant hormones including auxin, gibberellin (GA), ethylene (ETH), brassinosteroid (BR), and CK play very important roles in regulating cell elongation and division. GAs, BRs, and auxin can induce cell expansion [24]. In rice, the dwarf mutant *sd1* shows higher stature when activating GA signaling [33]. In rice and *Arabidopsis,* BR mutants display dwarfing phenotypes, showing that BRs determine stem elongation in monocotyledonous and dicotyledonous plants [34,35]. Many cytochrome P450 (CYP) genes are involved in BR biosynthesis [11]. In addition, many transcription factors (TFs) such as basic helix–loop–helix (bHLH), auxin response factors (ARF), ethylene response factor subfamily of AP2 (AP2/ERF), C2H2, and GRAS are important regulators of plant growth and development [36,37]. For instance, a number of bHLH transcription factors is involved in the regulation of cell elongation in response to BRs, GA, temperature, light, and developmental stages [36,37]. *AINTEGUMENTA* (*ANT*) encodes AP2/ERF family proteins, which can regulate integument cell division by prolonging cell proliferation time to control flower development-related cell division and organ size [20]. *JAGGED* (*JAG*) encodes a transcription factor protein with a C2H2 zinc finger domain. Partial loss of JAG function inhibits the development of lateral organs [38,39,40].

High-throughput profiling of transcripts or proteins is an efficient method to explore changes in complex biological processes [41]. For instance, a new model for light-induced anthocyanin biosynthesis was constructed through combined transcriptomic and proteomic analysis in eggplant (*Solanum melongena* L.) [42]. In petunias, RNA-seq and tandem mass tag (TMT) labeling proteomics were used to analyze the effect of ethylene on flower senescence, which offered an important resource for the functional analysis of Kub and facilitated the elucidation of the senescence process [43]. Transcriptome analysis was performed to reveal that the modification of cell wall biosynthesis, phytohormone biosynthesis, and signal transduction contributes to the dwarfing and narrow-leaf phenotype of the *dnl2* (*new dwarf and narrow-leaf*) mutant in maize [44].

In this study, we identified a spontaneous cucumber mutant that affects plant architecture including root, plant stature, stem diameter, internode, cotyledon, true leaf, male and female flowers, as well as fruit. Therefore, we named this mutant *organ development defective 1* (*odd1*). Through a series of physiological and morphological indices, it was found that the cell size and cell number of the cotyledon in *odd1* are significantly smaller than those in wild type (WT), and similar differences are observed in functional leaf and petal. The SAM became smaller, and the parthenocarpy characteristics disappeared in the *odd1* mutant. Genetic analysis demonstrated that the phenotype of the *odd1* mutant is controlled by a single recessive nuclear gene. Therefore, *odd1* is valuable material for studying the regulatory mechanisms of growth and development in cucumber, even in Cucurbitaceae. Through bulk segregation analysis, *odd1* was located on chromosome 5. Furthermore, integrated profiling of the transcriptome and proteome indicated that many genes and proteins related to growth and development showed significantly different expression in *odd1*. Furthermore, one model of plant hormone signaling pathways and one model of shoot apical meristem maintenance differentiation in *odd1* were presented. These results not only expanded and deepened the insights into the molecular regulation of organ growth and development in cucumber, but also provided a foundation for cloning and further functional analysis of *ODD1*.

## 2. Results

### 2.1. Phenotypic and Physiological Characterization of odd1 Mutant

**Figure 1:**
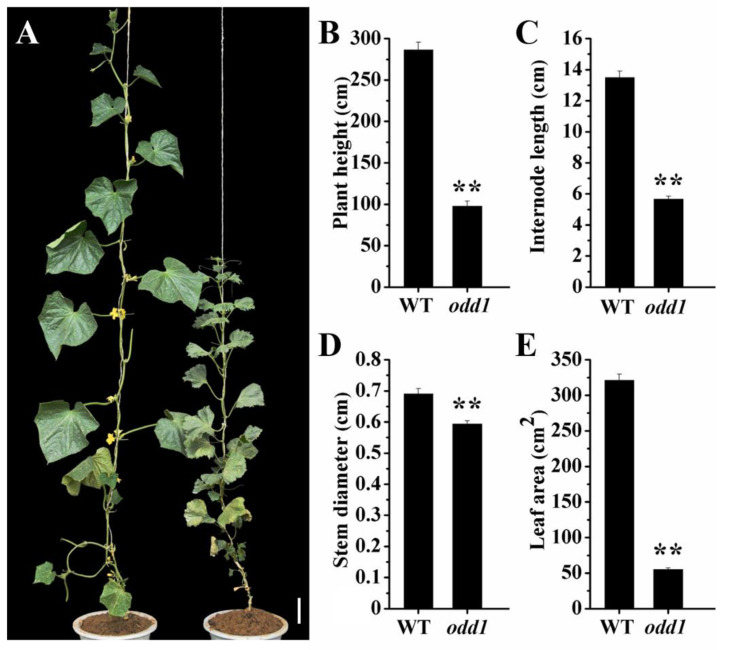
Phenotypic comparison between the WT and *odd1* mutant. (**A**) Adult plants of WT (left) and *odd1* (right), respectively. (**B**) Plant height. (**C**) Internode length. (**D**) Stem diameter. (**E**) Leaf area of WT and *odd1* at fruit setting stage. Scale bar represents 10 cm. ‘**’ indicates very significant differences at *p* = 0.01 level. Vertical bars represent standard deviation (n = 15).

**Figure 2:**
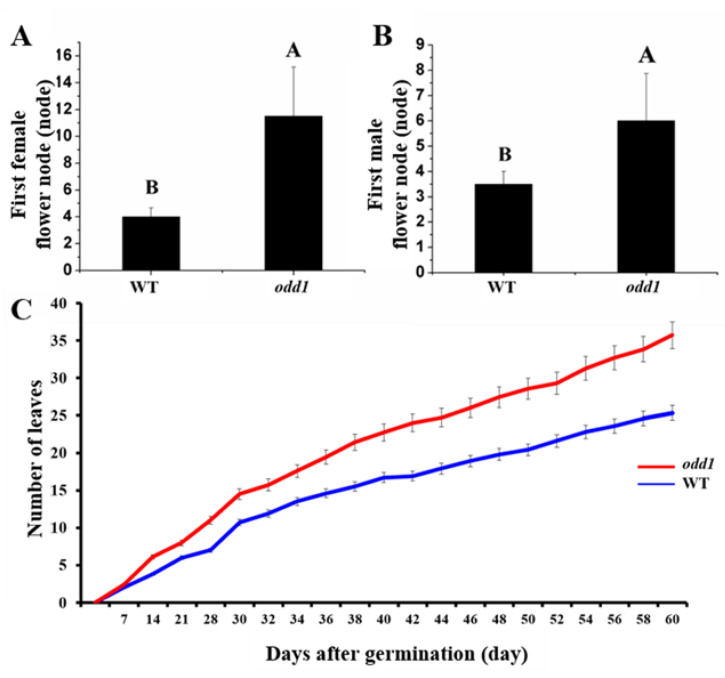
The comparison of first female and male flower position and leaf number between the WT and *odd1* mutant. (**A**) First female flower node of *odd1* and WT. (**B**) First male flower node of *odd1* and WT. (**C**) The leaf number at different developmental stages of *odd1* and WT. Different letters indicate very significant differences at *p* = 0.01 level. Vertical bars represent standard deviation (n = 15).

**Figure 3:**
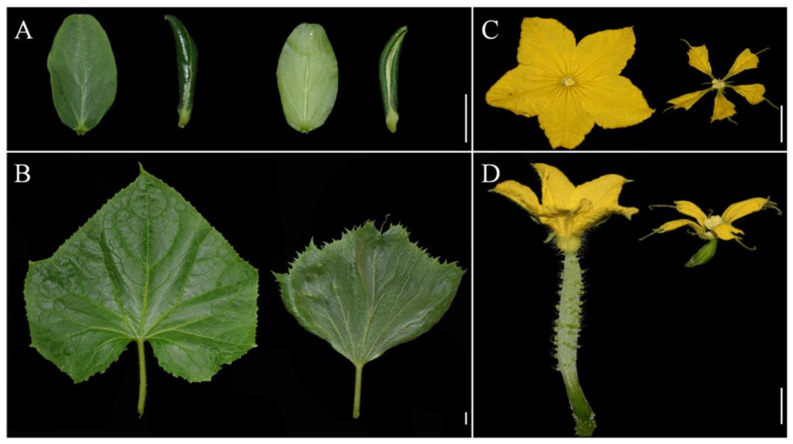
Phenotypic features of *odd1*. (**A**,**B**) The contrast of cotyledon and true leaf between WT (**left**) and *odd1* (**right**) at same developmental stage. (**C**) The male flower and (**D**) female flower at anthesis (left: WT; right: *odd1*). Scale bars represent 1 cm.

A spontaneous mutant was isolated from a North China type cucumber inbred line 09-1 (WT). Compared with WT, the mutant was found to have a much smaller plant stature (Figure 1A). At the adult stage, the average plant height of the WT was 286.67 cm, which was significantly higher than that of the mutant (97.83 cm) (Figure 1B). The internode length and the stem diameter of the mutant were drastically decreased (Figure 1C,D). The mutant true leaf was noticeably smaller than that of the WT (Figure 1A,E and Figure 3B). Observation of the entire growth cycle phenotype showed that compared with the WT, *odd1* significantly increased the node positions of the first female and male flowers. That is to say, flowering was delayed in *odd1 (*Figure 2A,B). Interestingly, during the same growth period, *odd1* had more leaves than WT (Figure 2C).

**Figure 4:**
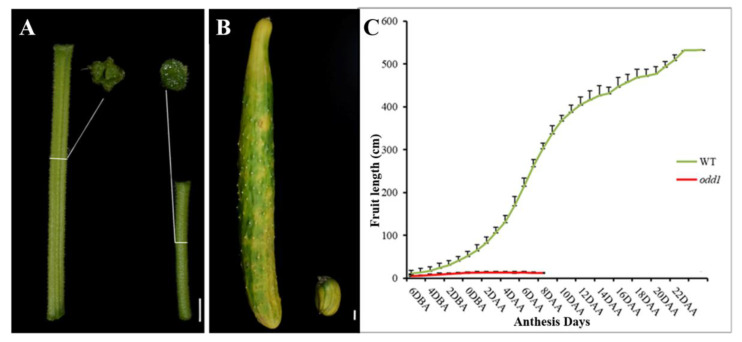
Phenotypic features of stem and fruit of *odd1*. (**A**) The contrast of stems and their transverse sections between WT (left) and *odd1* (right) at same developmental stage. (**B**) The mature fruits of WT (left) and *odd1* (right), respectively. (**C**) Fruit growth curves of *odd1* and WT. DBA. Days before anthesis. DAA. Days after anthesis. Scale bars represent 1 cm.

**Figure 5:**
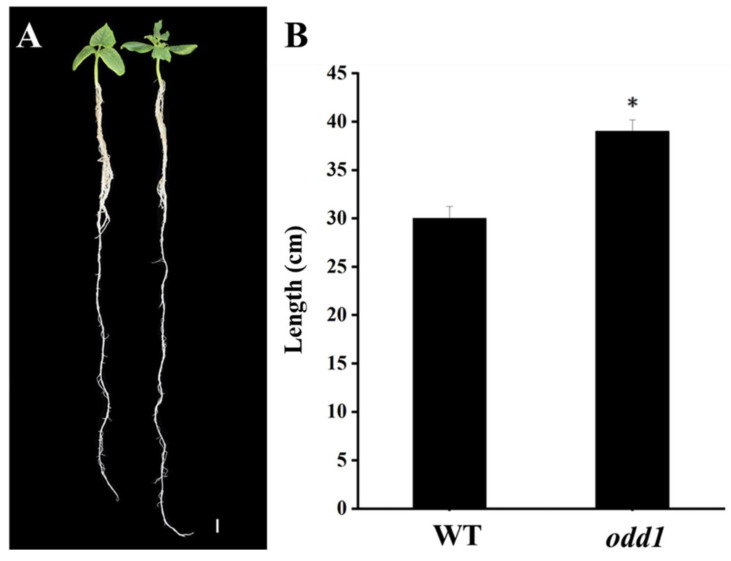
Phenotypic features of the root of *odd1*. (**A**) The contrast of roots between WT (**left**) and *odd1* (**right**) at 21 days after germination. (**B**) The quantification of root length in (**A**). Scale bars represent 1 cm. ‘*’ indicates very significant differences at *p* = 0.05 level. Vertical bars represent standard deviation (n = 10).

Further observation revealed that the mutant had dark green and curled-edge cotyledons (Figure 3A). Its true leaves with abnormal veins were wrinkled and dark green. Not only was the leaf size smaller, but also the leaf shape became like a ginkgo leaf (Figure 3B). The petiole of *odd1* was cylindrical (Figure 3B). The petals of male and female flowers of the mutant were deeply split and simultaneously smaller (Figure 3C,D). The stem of *odd1* was solid and cylindrical (Figure 4A). The mutant male flowers could produce normal pollen, but the female flowers were sterile, and only one fruit was observed on all the *odd1* mutant plants during the more than ten years of greenhouse and field cultivations, yet the fruit had no seeds and was significantly smaller and shorter than that of the WT (Figure 4B). We recorded the fruit growth curves of WT and *odd1* from 6 days before anthesis to 22 days after anthesis (Figure 4C). The average fruit length of *odd1* was 5.07 cm at 6 days before anthesis. On the 4th day after anthesis, the fruit of *odd1* grew to a maximum length of 12.91 cm. From the 5th day after anthesis to the 8th day after anthesis, the fruit of *odd1* began to shrink from 12.55 cm to 11.94 cm. The mutant fruit became withered from the 8th day after anthesis. The *odd1* mutant could clearly not set fruit (Figure 4C). Therefore, the *odd1* mutant is an ideal material to study the mechanism of fruit setting of cucumber. However, the root system of *odd1* was significantly longer (39 cm) than that of the WT (30 cm) at 21 days after germination (Figure 5).

Because the leaves of *odd1* seemed dark green, we wanted to know whether its chlorophyll content was higher. Then, we measured chlorophyll content and several photosynthetic parameters in WT and *odd1* and found that the chlorophyll and carotenoid contents were lower in the leaves of *odd1* than in those of WT (Table 1), suggesting that the dark green leaf color of *odd1* was not associated with higher chlorophyll content. In addition, the net photosynthetic rate (Pn), stomatal conductance (gs), and intercellular CO_2_ concentration (Ci) significantly decreased in *odd1*, but there was no significant difference in the transpiration rate (Tr) between WT and *odd1* (Table 1). The total chlorophyll content was significantly decreased in the *odd1* mutant and the transpiration rate (Tr) was similar between the WT and *odd1* mutant, which may suggest that the decreased photosynthesis in *odd1* is mainly related to the chlorophyll and/or light absorption capacity.

### 2.2. Histological and Anatomical Features of odd1 Mutant

**Figure 6:**
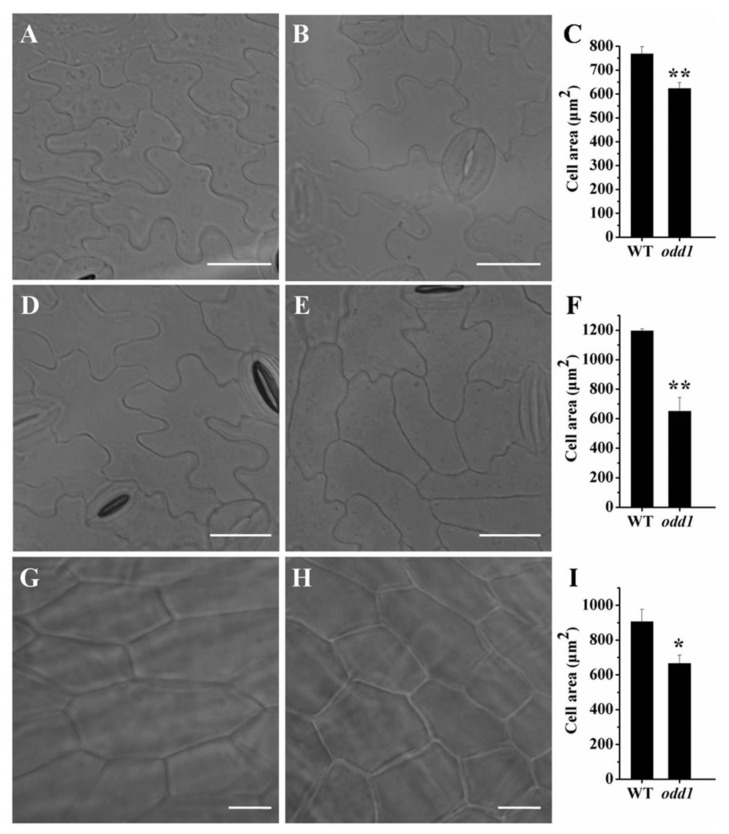
Comparison of the cell morphology between WT and *odd1*. (**A**–**C**) The cell shape and size of the cotyledon between WT (**A**) and *odd1* (**B**), and the corresponding quantifications of cell size (**C**). (**D**–**F**) The cell phenotype of the fourth functional leaf counting from the plant apex of WT (**D**) and *odd1* (**E**), and the respective quantifications of cell size (**F**). (**G**–**I**) Petal cells at anthesis of WT (**G**) and *odd1* (**H**), and the respective quantifications of cell size (**I**). Asterisk indicates that the cell size in WT is significantly larger than that in *odd1* (single asterisk, *p* = 0.05; two asterisks, *p* = 0.01). The bars in (**C**,**F**,**I**) represent the standard deviation (n = 6). Scale bars represent 20 μm.

To ascertain the cellular base of the organ size in *odd1*, we compared the epidermal cell size of the cotyledon, functional leaf, and petal in WT (Figure 6A,D,G) and *odd1* (Figure 6B,E,H). The cell size of the cotyledon in *odd1* was significantly smaller than that in WT (Figure 6C), and similar differences in functional leaf and petal were observed between WT and *odd1* (Figure 6F,I). The epidermal cell shape of the cotyledon and functional leaf were irregular in WT (Figure 6A,D), but the irregularities of the epidermal cells were significantly decreased in *odd1* (Figure 6B,E). The cell sizes of the cotyledon, functional leaf, and petal of *odd1* were 81.12%, 54.46%, and 73.42% of those of WT, respectively (Figure 6C,F,I); however, the areas (fifteen biological replicates) of the cotyledon, functional leaf, and petal of *odd1* were only 41.75% (278.63 mm^2^/667.38 mm^2^), 17.24% (55.43 cm^2^/321.56 cm^2^), and 16.27% (272.16 mm^2^/1672.77 mm^2^) of those of WT, respectively (Figure 1E and Figure 3). Therefore, the organ size decrease of *odd1* was due to the decrease of cell size and cell number.

**Figure 7:**
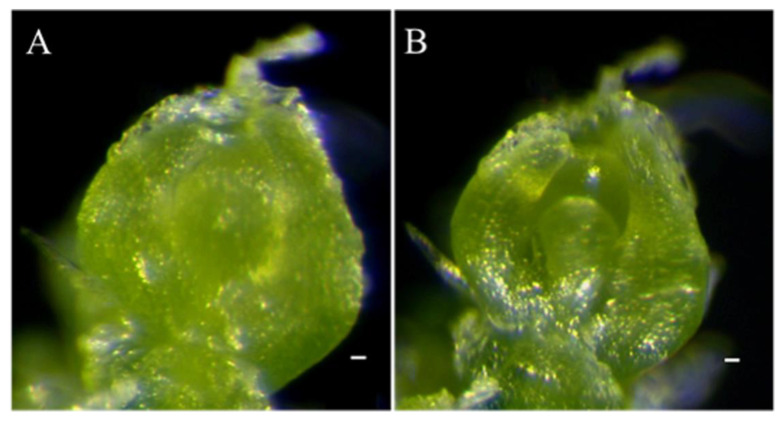
The SAM of WT and *odd1*. (**A**) WT. (**B**) *odd1.* Scale bars represent 10 μm.

SAM is the fundamental source of all aboveground organs including stem, leaf, flower, and fruit. To investigate the SAM of *odd1*, we compared the morphology of the SAM of *odd1* and WT using the 25-day-old seedlings. Compared with WT, *odd1* had a smaller SAM (Figure 7). Thus, the smaller aboveground organs of *odd1* might also be related to its smaller SAM.

### 2.3. The Female Sterility of odd1

**Figure 8:**
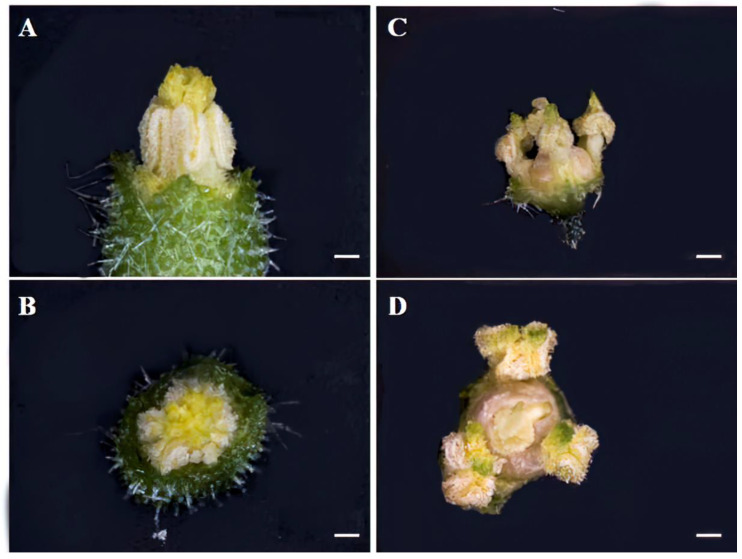
Anatomical observations of the stamen of *odd1*. (**A**,**B**) The stamen of WT at anthesis. (**C**,**D**) The stamen of *odd1* at anthesis. Scale bars represent 5 mm.

**Figure 9:**
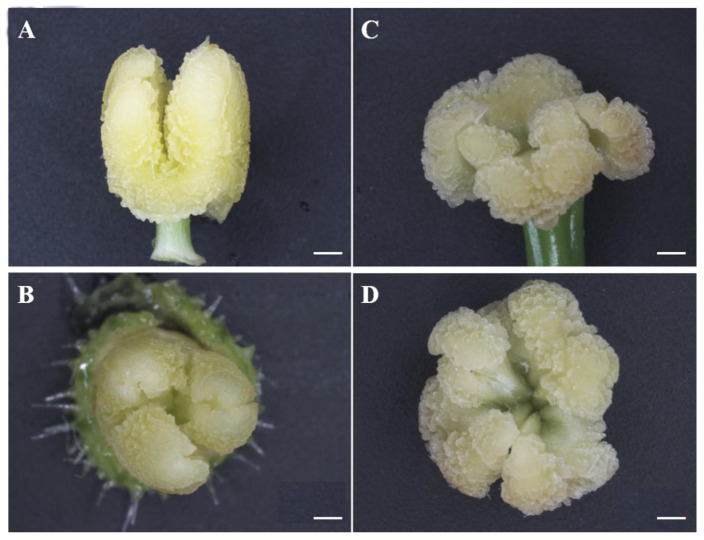
Anatomical observations of stigma of *odd1*. (**A**,**B**) The stigma of WT at anthesis. (**C**,**D**) The stigma of *odd1* at anthesis. Scale bars represent 5 mm.

Because the *odd1* mutant could not set fruit (Figure 4C), we observed carefully the female and male flowers of WT and *odd1* under a stereomicroscope. The stamen was split into three independent anthers in *odd1* compared to WT (Figure 8). Not only did the stigma became larger and valgus, but also the shape became cauliflower-like, and the style became thicker in *odd1* (Figure 9).

**Figure 10:**
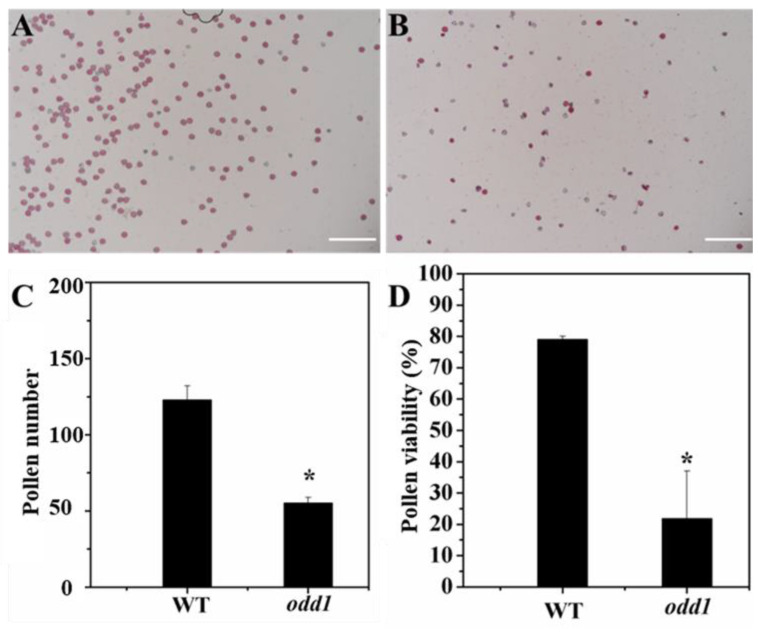
Pollen quantity and vitality of WT and *odd1*. (**A**,**B**) Pollen viability of WT and *odd1*. (**C**) The respective quantifications of pollen number in a single field of view. (**D**) The respective quantifications of vital pollen grains. Asterisk indicates significant differences (single asterisk, *p* = 0.05). The bars in C and D represent the standard deviation (n = 6). Scale bars represent 200 μm.

**Figure 11:**
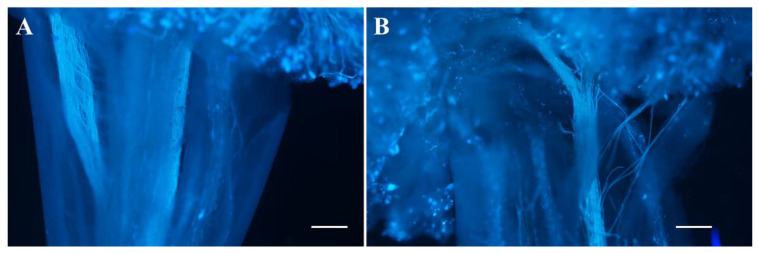
Fluorescence microscopic observation of pollen germination and pollen tube growth after pollination. (**A**) WT. (**B**) *odd1*. Scale bars represent 200 μm.

In addition to the morphologies of the stamen and pistil of *odd1*, we examined their fertilities. All fresh pollen grains of each flower were stained with Alexander solution, and the number and viability of pollen grains were observed and counted. The number of pollen grains of *odd1* was significantly less than that of WT (Figure 10A–C). After staining, the pollen grains with high vitality were purple or red, while those with no vitality were blue or withered. It can be seen from Figure 10A,B,D that *odd1* could produce normal and vital pollens, but its vital pollen percent was significantly lower than that of the WT. The pollens of WT and *odd1* were pollinated on the stigma of WT, respectively, to test pollen viability. Furthermore, the materials were stained with aniline blue 24 h after pollination. The pollen germination and pollen tube elongation were observed with a fluorescence microscope. The pollen grains of both WT and *odd1* could germinate normally and pass through papilla cells to form pollen tubes, which further indicated that the pollen of *odd1* was fertile (Figure 11). By reciprocal cross test and self-pollination, the WT plants could produce fruits with normal seeds (Table 2), while the *odd1* plants could not produce fruit (Figure 4C and Table 2). When WT was used as the female parent, seeds were normally produced in the fruit (Table 2), but when *odd1* was used as the female parent, seeds were not successfully produced (Table 2). Therefore, it was concluded that the *odd1* mutant was a female sterile mutant.

**Figure 12:**
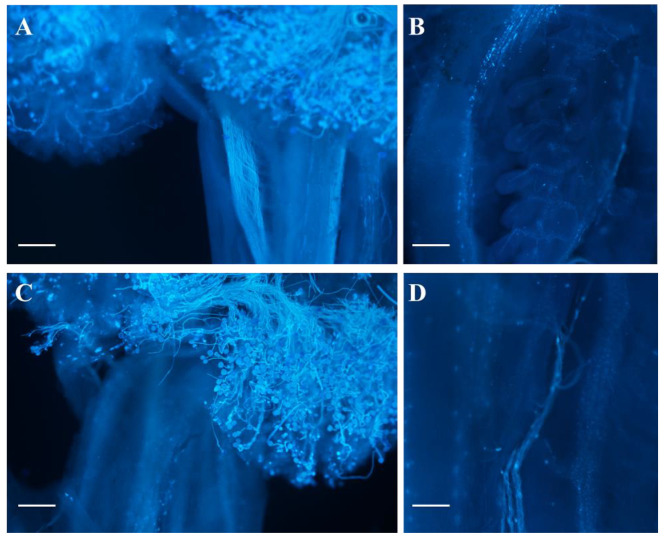
Fluorescence microscopic observation of pollen germination and pollen tube growth after pollination. (**A**,**B**) WT. (**C**,**D**) *odd1*. Scale bars represent 200 μm.

When pollens of WT were pollinated on the stigmas of *odd1* and WT, respectively, most pollen grains could germinate normally and pass through papilla cells to form pollen tubes, and the pollen tubes could enter the ovary through the stigma and style (Figure 12A,C). We further observed the pollen tubes in the ovary and failed to find ovules in the ovary of *odd1* (Figure 12B,D). To sum up, the reason for female infertility of *odd1* may be due to the absence of ovules in the mutant.

### 2.4. Genetic Characteristic and Mapping of odd1 Mutant

**Figure 13:**
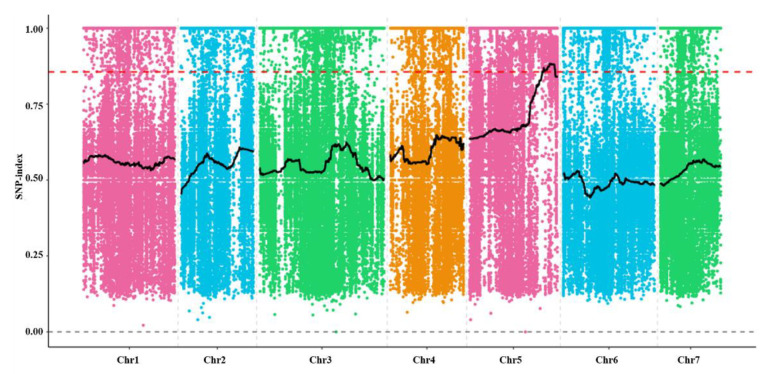
The association analysis of the SNP-index.

Three populations were developed for investigating the law of inheritance and mapping and cloning of *odd1* (Table 3). First, *odd1* was crossed with ‘Chinese long’ 9930 that has a similar phenotype with the WT to produce F_1_. All F_1_ progenies presented the WT phenotype. Then, an F_2_ population was obtained by F_1_ selfing. In the F_2_ segregating population, 57 of 200 exhibited the mutant phenotype (*χ*^2^ = 1.127 < *χ*^2^ _0.05, 1_ = 3.841; *p* > 0.05); in addition, a test cross (F_t_, F_1_ was crossed with *odd1* again) yielded 200 descendants comprising 97 WT and 103 mutant individuals (*χ*^2^ = 0.125 < *χ*^2^_0.05, 1_ = 3.841; *p* > 0.05), which fit the segregation ratios of 3:1 and 1:1, respectively. These results demonstrate that the phenotype of *odd1* mutant is controlled by a single recessive nuclear gene. Using the MutMap strategy, the *odd1* pool in the F_2_ population was directly subjected to genome resequencing, and then the genomic resequencing data were compared with the genome sequence of ‘Chinese long’ 9930 (Table S1). After filtering, 179,050 SNPs were used for association analysis. SNP-index correlation analysis was performed, and the *odd1* gene was successfully located on chromosome 5 with a physical distance of about 4.05 Mb (23.27–27.32 Mb) (Figure 13). Furthermore, the *odd1* gene is currently being fine-mapped.

### 2.5. The Transcriptomic and Proteomic Profiles of odd1 Mutant

To explore the molecular mechanism underlying the abnormal growth and development of organs in *odd1*, we performed transcriptomic and proteomic analysis using apical buds with growth points and apical leaves of 23-day-old seedlings from mutant and WT groups.

High-throughput RNA sequencing (RNA-seq) generated 42.78 to 63.66 million single-ended reads for each sample, and three biological replicates were performed for each group (Table S2). After low quality regions and adapter sequences being removed, 38.55–59.12 (93.72–94.49%) million clean reads were mapped to the cucumber genome (http://cucurbitgenomics.org/, accessed on 20 December 2021) and combined with known gene annotations (http://cucurbitgenomics.org/organism/2, accessed on 20 December 2021) using Hisat2 (v2.0.4) (Table S2). Furthermore, 93.1–94.2% of clean reads were mapped to the annotated genes in the reference genome (Table S3). We summarized the expression level of each gene with HTSeq (v0.9.1); 24,118 genes were obtained from the six samples. Pearson correlation analysis showed high repeatability and reliability (R^2^ > 0.945) among three replicates (Figure S1A). Furthermore, principal component analysis displayed clear separation between WT and *odd1* mutant groups (Figure S2A). Genes with adjusted *p*-values < 0.05 found by the DESeq R package (1.18.0) were assigned as differentially expressed genes (DEGs) between WT and mutant groups. We found 565 DEGs, in which 314 genes were significantly up-regulated, and 251 genes were significantly down-regulated in the mutant group compared to WT (Table 4). In order to validate the RNA-seq data, the expression levels of some DEGs were evaluated by qRT-PCR (quantitative RT-PCR) (Figure S3).

To examine the proteins altered by *odd1*, proteomic profiles were analyzed between mutant and WT groups with three biological replicates for each group. In total, 33,749 peptides, 31,738 unique peptides, and 6283 protein groups were identified, among which 5456 proteins were quantified (Table 4). Pearson correlation analysis showed high repeatability and reliability (R^2^ > 0.95) among three biological replicates (Figure S1B). Furthermore, principal component analysis displayed clear separation between the WT and *odd1* mutant (Figure S2B). A total of 356 differentially expressed proteins (DEPs) was observed, of which 176 proteins were up-regulated, and 180 proteins were down-regulated in the mutant group compared to the WT group with a threshold of 1.2-fold and a *p*-value < 0.05 (Table 4).

### 2.6. GO Analysis of DEGs and DEPs

**Figure 14:**
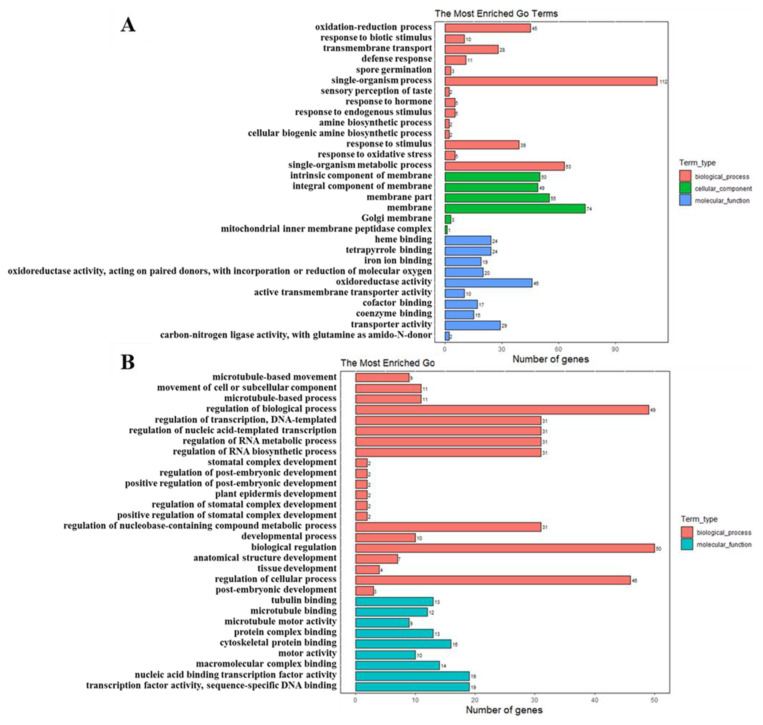
GO enrichment analysis of DEGs. (**A**) GO enrichment analysis of significantly up-regulated genes. (**B**) GO enrichment analysis of significantly down-regulated genes.

The goal of the GO (Gene Ontology) consortium is to produce a dynamic, controlled vocabulary that can be applied to all eukaryotes, even as knowledge of gene and protein roles in cells is accumulating and changing. To further determine the function of these DEGs, GO term enrichment analysis was performed with an adjusted *p*-value < 0.05, and the top 30 GO terms with the most significant enrichment are shown in Figure 14A,B. The results covered a wide range of biological processes, cellular components, and molecular functions (Figure 14A,B). For the up-regulated genes in *odd1*, the term with the most DEGs was the ‘single-organism process’ (112 genes) in the biological process group (red in Figure 14A). Notably, a large portion of resistance-related terms including ‘oxidation–reduction process’, ‘response to biotic stimulus’, ‘defense response’, ‘response to endogenous stimulus’, ‘response to stimulus’, and ‘response to oxidative stress’ were also significantly enriched in the biological process group (red in Figure 14A). Interestingly, the significantly enriched GO terms in the cellular component group were all related to membrane, such as ‘membrane’, ‘membrane part’, and ‘integral component of membrane’ (green in Figure 14A). ‘Oxidoreductase activity’ was the category with the most DEGs in the molecular function group (blue in Figure 14A). For the genes that were down-regulated in *odd1*, the analysis of the biological process showed that ‘regulation of biological process’ (49 genes) was the top DEGs term (red in Figure 14B). Moreover, many development-related DEGs were significantly enriched in the biological process as follows: ‘stomatal complex development’, ‘regulation of post-embryonic development’, ‘positive regulation of post-embryonic development’, ‘plant epidermis development’, ‘regulation of stomatal complex development’, ‘positive regulation of stomatal complex development’, ‘developmental process’, ‘anatomical structure development’, ‘tissue development’, and ‘post-embryonic development’ (red in Figure 14B). In terms of molecular function, the top two DEGs terms were ‘transcription factor activity, sequence-specific DNA binding’ and ‘nucleic acid binding transcription factor activity’ (blue in Figure 14B). In addition, many of microtubule-related DEGs were highly enriched in the biological process or molecular function for the following: ‘microtubule-based movement’, ‘microtubule−based process’, ‘tubulin binding’, ‘microtubule motor activity and microtubule binding’ (Figure 14B). In the top 30 GO terms, no significantly enriched DEGs were detected in the molecular function group. These results suggest that *odd1* likely promotes many resistance-related processes while inhibiting development-related and transcription factor activity processes.

**Figure 15:**
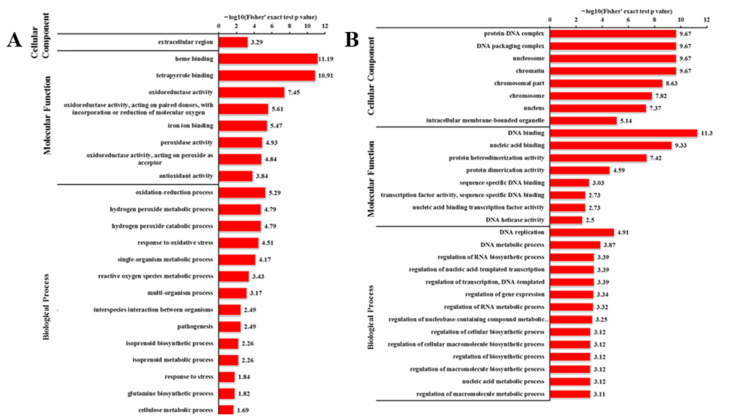
GO enrichment analysis of DEPs. (**A**) GO enrichment analysis of significantly up-regulated proteins. (**B**) GO enrichment analysis of significantly down-regulated proteins.

To elucidate the functional differences between the down-regulated and up-regulated proteins, the quantified proteins were analyzed for GO enrichment with an adjusted *p*-value < 0.05 (Figure 15A,B). In the cellular component category, the up-regulated proteins were enriched in the ‘extracellular region’ (4 proteins) (Figure 15A). In the molecular function category, most of the up-regulated proteins were involved in ‘oxidoreductase activity’ (38 proteins), ‘heme binding’ (19 proteins), and ‘tetrapyrrole binding’ (19 proteins) (Figure 15A). The analysis of biological processes showed that ‘single-organism metabolic process’ (51 proteins), ‘oxidation–reduction process’ (34 proteins), ‘response to oxidative stress’ (8 proteins), ‘hydrogen peroxide metabolic process’ (6 proteins), ‘hydrogen peroxide catabolic process’ (6 proteins), and ‘reactive oxygen species metabolic process’ (6 proteins) occupied a large proportion of the up-regulated proteins (Figure 15A). In terms of down-regulated proteins, the top two DEPs terms were ‘intracellular membrane-bounded organelle’ (19 proteins) and ‘nucleus’ (17 proteins) in the cellular component (Figure 15B). The most significantly enriched GO terms were ‘DNA binding’ (26 proteins) and ‘nucleic acid binding’ (42 proteins) in the molecular function group (Figure 15B). In the biological process category, ‘nucleic acid metabolic process’ (14 proteins) was the top DEPs term (Figure 15B). These results imply that nucleic acid processing might be suppressed by the mutation of *odd1*, and these terms may play important roles for growth and development regulation in cucumber.

### 2.7. Transcription Factors Are Involved in Cucumber Growth and Development and Resistance Control

For *odd1*-changed transcription factor activity processes (blue in Figure 14B), we further analyzed the significantly differentially expressed transcription factors in the *odd1* mutant. A total of 31 transcription factors was found to be repressed in the apical bud with the growth point and apical leaf of *odd1*, and they were distributed in different gene families (Table S4). These 31 genes could be subdivided into nine groups, namely AP2-EREBP, C2H2, HSF, HB, WRKY, bHLH, GRAS, and MYB, and other transcription factor genes, which displayed lower expression in the *odd1* mutant with defects of growth and development, implying that these transcription factors may function as positive regulators in cucumber growth and development. By contrast, the expression of 37 transcription factors belonging to the following gene families was up-regulated in the *odd1* mutant: bHLH, bZIP, HB, MYB, ARF, WRKY, NAC, C2H2, LOB, TCP, AUX/IAA, and others (Table S5). Notably, *Csa1G042780*, the homologous gene of *WOX1* (*WUSCHEL-related homeobox 1*), which plays a positive role in lateral organ formation in *Arabidopsis* [45] and petunia [46], showed a 2.25-fold reduction in the *odd1* mutant (red in Table S4). In petunia, the mutation of the *WOX1* homologous gene lead to the abnormal development of pistils and longer and narrower petals and leaves [47]. CsWOX1 regulates leaf vein patterning and the development of leaf by CsPID-mediated auxin transport [15], and *CsWOX1* is very important for anther and pollen development of male flowers in cucumber [48]. It is speculated that *odd1* regulates the growth and development of organs by inhibiting the expression of *CsWOX1* in cucumber. Moreover, many of these transcription factor-encoding genes are known to function in plant growth and development, such as *Csa3G354520*, a homologous gene of the bHLH domain-containing transcription factor that is involved in the regulation of cell elongation in response to BRs, GA, temperature, light, and developmental stages [36,37]; *Csa6G495010* and *Csa3G405510* and *Csa6G040600,* the homologous genes of GRAS transcription factors that exert important roles in signal transduction, meristem maintenance, and development in *Arabidopsis* [49]; *Csa3G141860* and *Csa7G071440* and *Csa7G428260* and *Csa1G043020* and *Csa5G092930*, the homologous genes of C2H2 proteins which could be involved during all the stages of reproductive development from panicle initiation till seed maturation in indica rice [50] and that control carpel numbers and ovule development in *Arabidopsis* [51]; *Csa6G291920 and Csa6G518210 and Csa6G524670*, the homologous genes of ARF transcription factors which play important roles in auxin responsive transcription [52]; *Csa7G378520* and *Csa1G397130* and *Csa3G143570*, the homologous genes of AUX/IAA transcription factors which are necessary for auxin signal transduction [53,54]; *Csa4G629480* and *Csa1G038340* and *Csa3G824990*, the homologous genes of NAC transcription factors exert important roles in secondary cell wall biosynthesis [55], leaf senescence [56,57], and lateral root development [58,59]; *Csa1G020890* and *Csa1G033030*, the homologous genes of TCP transcription factors which participate in multiple growth processes including embryonic growth, leaf development, floral morphogenesis, cell cycle regulation, and hormone signal transduction [60]; six genes (*Csa3G826690*, *Csa1G033200*, *Csa1G042350*, *Csa7G413890*, *Csa6G040640*, and *Csa2G100550*), the homologous genes of MYB transcription factors which are involved in axillary meristem regulation, lateral organ formation, and shoot branch regulation in *Arabidopsis* [61]; and *Csa3G751470*, a homologous gene of GRF transcription factor that mainly acts as a positive regulator of cell proliferation involved in the regulation of stem and leaf development (Tables S4 and S5) [62]. These results suggest that these transcription factors might play critical roles in regulating cucumber growth and development.

Intriguingly, many resistance-related transcription factors were found significantly differentially expressed in *odd1* mutant including AP2-EREBP [63], C2H2 [64], HSF [65], WRKY [66], bHLH [67], MYB [68], bZIP [69], and NAC [70]. For example, *Csa7G428890*, a homologous gene of the bHLH122 transcription factor that is important for drought and osmotic stress resistance in *Arabidopsis* [71]; *Csa2G416070* and *Csa5G642710*, the homologous genes of bZIP transcription factors that usually play positive roles in many abiotic and biotic stress responses such as hypersensitive response (HR), salicylic acid (SA), ethylene (ET), pathogen defense, and oxidative stress [69], showed 2.4- or 1.2-fold increases in the *odd1* mutant (Table S5); *Csa3G141860* and *Csa7G071440* and *Csa7G428260* and *Csa1G043020* and *Csa5G092930*, the homologous genes of C2H2 proteins which could be involved in different abiotic stress conditions such as low temperature, salt, drought, osmotic stress, oxidative stress, and the biotic stress signaling pathway [64]; and six genes (*Csa5G155570, Csa1G075060, Csa4G630010, Csa7G447150, Csa5G612310, Csa3G357110*, and *Csa6G091830*), the homologous genes of AP2/ERF transcription factors which are important regulators of drought, high salt, and low temperature stresses [72,73] (Tables S4 and S5). These results imply that *odd1* likely promotes many resistance-related processes by regulating the expression levels of these resistance-related transcription factors.

### 2.8. KEGG Pathway Analysis for DEGs and DEPs

**Figure 16:**
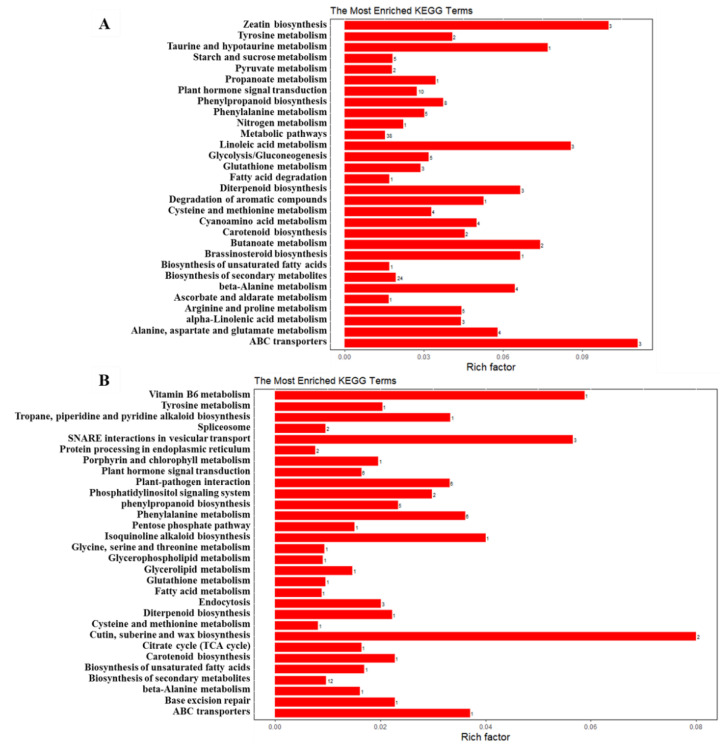
KEGG pathway analysis of DEGs. (**A**) KEGG pathway analysis of significantly up-regulated genes. (**B**) KEGG pathway analysis of significantly down-regulated genes.

**Figure 17:**
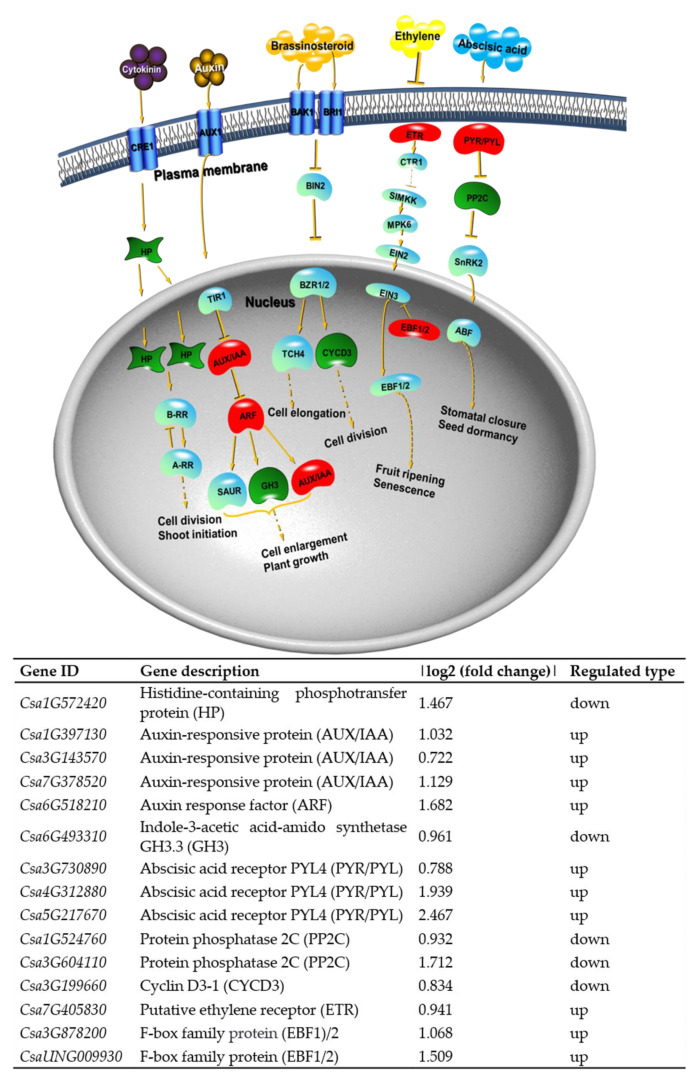
Schematic representation of significantly enriched pathways of plant hormone signal transduction in *odd1* vs. WT. Red shapes indicate up-regulation in *odd1* vs. WT; green shapes indicate down-regulation in *odd1* vs. WT; and blue shapes indicate no significant changes in *odd1* vs. WT.

**Figure 18:**
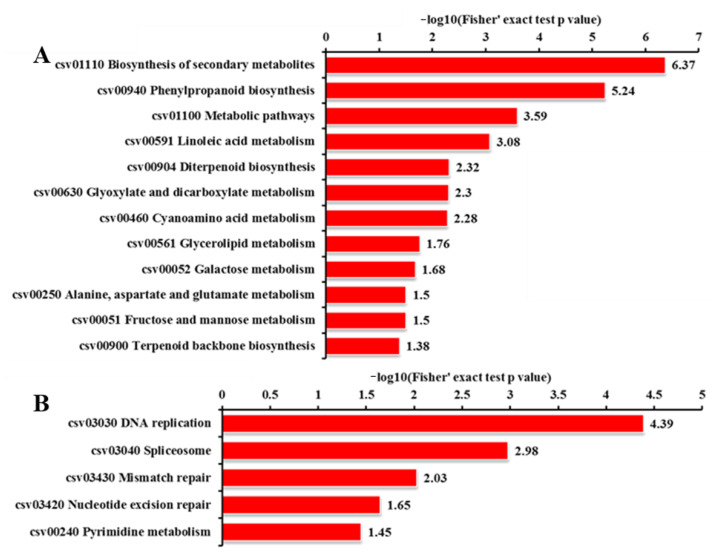
KEGG pathway analysis of DEPs. (**A**) KEGG pathway analysis of significantly up-regulated proteins. (**B**) KEGG pathway analysis of significantly down-regulated proteins.

We also performed KEGG (Kyoto Encyclopedia of Genes and Genomes) enrichment analysis for DEGs and DEPs with an adjusted *p*-value < 0.05 to reveal whether the growth and development-related genes were involved in specific pathways. The 565 DEGs were mapped to 60 KEGG pathways, and the top 20 KEGG terms with the most significant enrichment are shown in Figure 16. Among the up-regulated genes, the most enriched category was ‘ABC transporters’, followed by ‘zeatin biosynthesis’ and ‘linoleic acid metabolism’ categories (Figure 16A). Among the down-regulated DEGs, ‘cutin, suberine, and wax biosynthesis’, ‘vitamin B6 metabolism’, and ‘SNARE interactions in vesicular transport’ were significantly enriched. ‘Porphyrin and chlorophyll metabolism’, ‘carotenoid biosynthesis’, and ‘biosynthesis of unsaturated fatty acids’ were also highly enriched (Figure 16B). The pathway ‘plant hormone signal transduction’ was observed to occur among both up-regulated and down-regulated genes (Figure 16), suggesting the important role of *odd1* on hormone signal transduction in cucumber. The significantly influenced genes included *HP*, which is involved in cytokinin signaling (Figure 17); *AUX/IAA*, *ARF*, and *GH3*, which are involved in auxin signaling (Figure 17); *CYCD3*, which is involved in brassinosteroid signaling (Figure 17); *ETR* and *EBF1/2*, which are involved in ethylene signaling (Figure 17); and *PYR/PYL* and *PP2C*, which are involved in abscisic acid signaling (Figure 17). Among the up-regulated proteins, the top three enriched pathways were ‘biosynthesis of secondary metabolites’, ‘phenylpropanoid biosynthesis’, and ‘metabolic pathways’ (Figure 18A). Moreover, the down-regulated DEPs were involved in ‘DNA replication’, ‘spliceosome’, ‘mismatch repair’, ‘nucleotide excision repair’, and ‘pyrimidine metabolism’ (Figure 18B). These results indicate that *odd1* likely impacts many secondary metabolism processes. Consistent with GO analysis, nucleic acid processing was also suppressed in *odd1.*

### 2.9. Comparison of Transcriptome and Proteome Data

**Figure 19:**
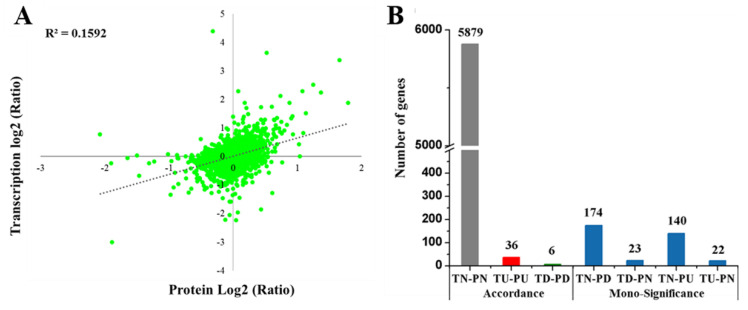
Combined transcriptome and proteome analysis of *odd1*. (**A**) The correlation analysis of abundance changes from transcriptome to proteome. (**B**) Comparison of significant differences between the transcriptome and proteome. T: transcript; P: protein species; N: no change; U: up-accumulation; D: down-accumulation.

We conducted a correlation analysis between the quantitative transcriptomic and proteomic data. A positive correlation of R^2^ = 0.1592 (R represents the Pearson correlation coefficient) was observed (Figure 19). Comparing proteomic and transcriptomic datasets, 6280 peptides or transcripts were identified both in the proteome and transcriptome (Figure S4). In more detail, 5921 members showed a consistent changing trend between the transcriptome and proteome, and 359 members showed mono-significant differences between the transcriptome and proteome. No member had opposite changing trends between the transcriptome and proteome (Figure 19B). The 87 of 565 DEGs from transcriptomic analysis had quantitative information for their respective proteins in the proteome. Forty-two genes (named as cor-DEG-DEP genes) were regulated at both the transcriptional (>1.5-fold and *p* < 0.05) and translational (>1.2-fold and *p* < 0.05) levels (Table 5). The 42 cor-DEG-DEP genes showed similar expression trends at the two levels (Figure 19B), and 36 of them were both significantly up-regulated genes (named as PU-TU genes), while another six genes were both significantly down-regulated (named as PD-TD genes) at two levels in the *odd1* mutant compared to WT.

### 2.10. Analysis of the 42 Cor-DEG-DEP Genes

**Figure 20:**
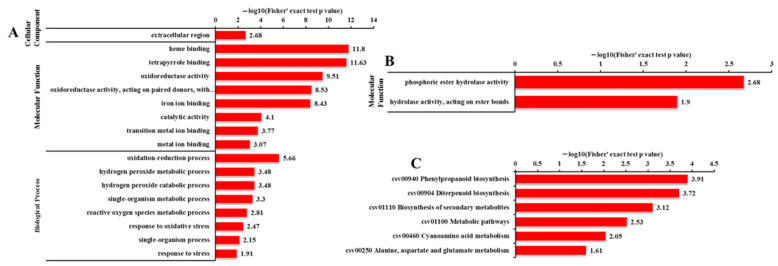
GO and KEGG analyses of the 42 cor-DEG-DEP genes. (**A**) GO enrichment analysis of PU-TU genes. (**B**) KEGG pathway analysis of PU-TU genes. (**C**) KEGG pathway analysis of PD-TD genes. T: transcript; P: protein species; N: no change; U: up-accumulation; D: down-accumulation.

We performed GO term and KEGG enrichment analysis for the 42 cor-DEG-DEP genes (adjusted *p*-value < 0.05). For the PU-TU genes in *odd1*, ‘extracellular region’ was the only significantly enriched GO term in the cellular component group (Figure 20A). In the molecular function category, the up-regulated proteins were enriched in ‘heme binding’, ‘tetrapyrrole binding’, ‘oxidoreductase activity’, ‘oxidoreductase activity, acting on paired donors, with incorporation or reduction of molecular oxygen’, ‘iron ion binding’, ‘catalytic activity’, ‘transition metal ion binding’, and ‘metal ion binding’. ‘Oxidation–reduction process’, ‘hydrogen peroxide metabolic process’, ‘hydrogen peroxide catabolic process’, ‘single-organism metabolic process’, ‘reactive oxygen species metabolic process’, ‘response to oxidative stress’, ‘single-organism process’, and ‘response to stress’ were the highly enriched terms in biological process (Figure 20A). These genes were enriched in ‘phenylpropanoid biosynthesis’, ‘diterpenoid biosynthesis’, ‘biosynthesis of secondary metabolites’, ‘metabolic pathways’, ‘cyanoamino acid metabolism’, and ‘alanine, aspartate, and glutamate metabolism’ pathways (Figure 20B). Two PD-TD genes were enriched both in ‘phosphoric ester hydrolase activity’ and ‘hydrolase activity, acting on ester bonds’ in the molecular function (Figure 20C). Interestingly, among the PU-TU genes, a GA-regulated gene (*Csa3G872170*) showed a 2.4-fold change, a gene (*Csa5G576590*) was related to auxin efflux, and eight genes were related to cytochrome P450s (CYPs) (Table 5). We suggest that these genes and related pathways might play important roles in cucumber growth and development.

## 3. Discussion

Developmental mutants are ideal materials in the study of cucumber growth and development. Although several types of developmental mutants have been described in cucumber, most mutations only affect a single phenotype or organ. For example, mutation of the *CsDET2* gene leads to the dwarf phenotype in cucumber [12]. The shortened fruit of *sf2* is caused by the mutation of *Histone Deacetylase Complex 1*, which directly regulates hormone synthesis and signal transduction-related genes [18]. In this study, *odd1* is a spontaneous cucumber mutant caused by a single recessive mutation, which largely affects the growth and development of the whole plant. The *odd1* mutant has a much smaller plant stature, longer root system, decreased internode length and stem diameter, solid stem, smaller dark green and curled-edge cotyledons, smaller wrinkled and simultaneously ginkgo leaf-like true leaves, smaller and split petals of male and female flowers, split anthers, larger valgus and simultaneously cauliflower-like stigma, thicker style, delayed flowering, more leaves, as well as disappeared ovules (Figure 1, Figure 2, Figure 3, Figure 4, Figure 5, Figure 6, Figure 7, Figure 8, Figure 9, Figure 10, Figure 11 and Figure 12). All the above data indicate that the function of *odd1* is very powerful in cucumber growth and development. Therefore, *odd1* is an ideal material for studying the regulatory mechanisms of growth and development in cucumber, even in Cucurbitaceae.

The ability to regulate growth and development is important for improving planting density and productivity in cucumber breeding. We combined phenotypic and anatomical observations, physiological and cytological analyses, and an integrated profiling of the transcriptome and proteome in order to explore the possible regulation mechanisms underlying the mutant phenotype of *odd1*. Our results demonstrated that the decrease of cell size and cell number, and the shorter and narrower SAM in *odd1* compared with the WT, could be the direct cause of the size decrease and the abnormal growth and development of organs in odd1 (Figure 6 and Figure 7). Through a series of comprehensive analyses, such as anatomical observation, pollen vigor identification, cross test, and observation of pollen germination in vivo, it was found that the pollen grains in *odd1* plants was fertile, and the pollen quantity and viability decreased, but the female flowers were sterile. Further studies have shown that the main reason for female sterility of *odd1* may be due to the absence of ovules in the mutant (Figure 8, Figure 9, Figure 10, Figure 11 and Figure 12).

Cucumber is an allogamous plant that can display heterosis. When crossed with female lines, the use of female sterile lines may be a very efficient way to produce hybrid seeds, since there is no need to manually isolate the flowers, which reduces the cost and improves the rate of hybrid seed production. Therefore, *odd1* may be a useful female sterile line for heterosis breeding in the future. The development of male and female organs is closely related to sex differentiation and fruiting. The female sterile mutants reported so far mainly include partially female sterile [74] and male and female sterile [75,76,77,78]; *odd1* can produce normal pollen, but female flowers are sterile, which is different from the above mutants. Most pollen grains of *odd1* can germinate normally and pass through papilla cells to form pollen tubes, and the pollen tubes can enter the ovary through the stigma and style (Figure 11), but we failed to find ovules in the ovary, and fertilization cannot be completed (Figure 12). This is different from the previous common causes of female sterility, such as abnormal structure of female flowers [79], absence of a normal embryo sac, or abnormal endosperm [80]. More evidence is needed to better understand the reason and the regulation mechanism of female sterility in *odd1*.

**Figure 21:**
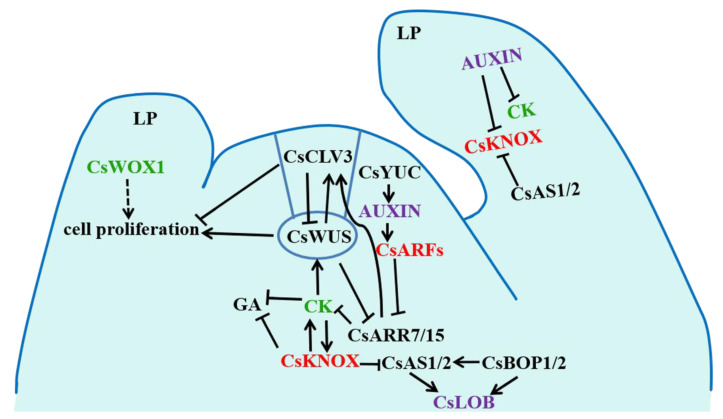
Model diagram of expression changes of growth and developmental regulatory genes and proteins in *odd1* vs. WT. Red indicates up-regulation in *odd1* vs. WT; green indicates down-regulation in *odd1* vs. WT; and purple indicates both up-regulated and down-regulated genes in *odd1* vs. WT.

In a deeper sense, a model that explores the molecular mechanisms underlying the abnormal growth and development of organs in the SAM of *odd1* was presented (Figure 21). It has been well demonstrated that *WOX* family members play positive roles in key developmental processes in plants, such as embryonic patterning, stem cell maintenance, and organ formation by promoting cell division activity and preventing premature cell differentiation [31,32]. *CsWOX1* showed a 2.25-fold reduction in the *odd1* mutant (red in Table S4). The significantly decreased expression of *WOX1* provides a possible molecular explanation for the dramatic reduction in cell size and number, some of the main reasons for the defects of growth and development in *odd1*. In cucumber, CsWOX1 regulates leaf vein patterning and the development of leaf by CsPID-mediated auxin transport [15], and *CsWOX1* is very important for anther and pollen development of male flowers [48]. In *Arabidopsis*, *WOX1* plays a positive role in lateral organ formation [45]. In petunia, the mutation of the *WOX1* homologous gene lead to the abnormal development of pistils and longer and narrower petals and leaves [47]. It is speculated that the repression of *CsWOX1* expression might be the reason for the growth and development defects in *odd1*. Moreover, the hormone-related genes were differentially expressed between *odd1* and WT, including *HP* involved in CK signaling; *CYCD3* involved in BR signaling; and *AUX/IAA*, *ARF*, and *GH3* involved in auxin signaling, which play critical roles in regulating cell elongation and division [24]. In particular, *HP* (*Csa1G572420*), a cucumber orthologue of *Arabidopsis* histidine phospho transfer proteins (AHPs), which are a critical component of plant CK signaling [19], was significantly repressed in the *odd1* mutant (Figure 17). A previous study suggested that AHPs function positively in CK signaling except AHP428 [81]. Mutations in these positive AHPs can result in developmental defects [82]. Moreover, *CYCD3* (*Csa3G199660*), a D-type plant cyclin gene through which CK activates cell division [58], was significantly repressed in the *odd1* mutant. In *Arabidopsis*, the promotion effect of BRs on cell division involves a distinct CYCD3-induction pathway [58]. In addition, BRs are steroid hormones that play essential roles in cell elongation, male fertility, senescence, and xylem differentiation [83,84]. Therefore, the significantly decreased expression of *HP* and *CYCD3* in *odd1* may serve as another main reason for the defects of growth and development in *odd1*. At the same time, many other TFs such as bHLH [36,37], C2H2 [38,39,40], GRAS [36,37], MYB [68], ARF [35,36], AUX/IAA [24], NAC [24], TCP [85], and GRF [24] identified in this study were also reported to affect growth and development. However, the relationship between these TFs and defects of growth and development in cucumber needs further experimental validation. In addition, one GA-regulated gene (*Csa3G872170*), one auxin efflux-related gene (*Csa5G576590*), and eight CYP450-related genes (*Csa3G903550*, *Csa6G088160*, *Csa3G698490, Csa5G224130, Csa3G903540, Csa6G088710, Csa1G044890*, and *Csa6G088170*), which were altered at both the transcriptional and translational levels (Table 5), were reported to participate in the regulation of growth and development [11,24]. In conclusion, the significantly decreased expression of *CsWOX1*, *CsHP*, and *CsCYCD3*, and the altered expression of other genes and hormones and TFs and proteins related to development might be the main reasons for the abnormal growth and development of organs in *odd1* (Figure 21).

Plant defenses to biotic and abiotic stresses are costly and often accompanied by significant growth inhibition. Increasing evidence demonstrates the potential trade-off involved in resistance and growth [86,87]. In this study, an integrated profiling of the transcriptome and proteome revealed that *odd1* likely promotes many resistance-related processes while inhibiting development-related processes because of many genes and proteins related to stresses altered in *odd1* (Figure 14, Figure 15, Figure 16, Figure 18 and Figure 20 and Table S5). Our preliminary observation showed that the waterlogging tolerance of *odd1* is enhanced. Further studies of waterlogging tolerance in *odd1* are ongoing. At the same time, the defects of growth and development were also observed in *odd1*. These results suggest that *odd1* might play a key regulatory role in balancing growth and development and resistance in cucumber.

In summary, the results of the present study demonstrated that the *odd1* gene is a pleiotropic effector of growth and development and might be a regulator in stress responses in cucumber. Future studies, such as cloning and functional analysis of *odd1*, are needed to better understand the molecular mechanisms involved in the regulation of cucumber stress resistance and growth and development.

## 4. Materials and Methods

### 4.1. Plant Materials and Growth Conditions

Cucumber (*Cucumis sativus* L., 2n = 14) North China inbred line 09-1 (WT), ‘Chinese long’ 9930, and the *odd1* mutant were used. Plants were grown with appropriate management for two generations each year from 2013 to 2022 in the standard greenhouse of the experimental field at Shandong Agricultural University. Eight to ten apical buds with growth points and apical leaves of 23-day-old seedlings from different plants were pooled together as one biological sample for the WT or mutant group for transcriptomic and proteomic analyses. Samples were immediately frozen in liquid nitrogen and stored at −80 °C until further use. All experiments were conducted at least three times with independently collected and extracted tissues unless noted otherwise.

### 4.2. Phenotypic Characterization and Photosynthesis-Related Parameters in the WT and odd1 Mutant

Thirty mutant and WT plants were grown in the greenhouse. At the fruit setting stage (seventy-day-old), the vine length, width of the stem, number of nodes and internode length, and leaf area (the fourth functional leaf from the top) were measured. Each parameter was determined from 15 biological repeats. Phenotypes of the cucumber plants were recorded using an optical camera (D7100, Nikon, Japan).

We measured chlorophyll content and several photosynthetic parameters in mutant and WT plants. Leaf gas exchange was measured with a Ciras-3 Portable Photosynthesis System (PP-Systems company, Amesbury, MA, USA) on the fourth functional leaves from the top at the fruit setting stage under 1000 μmol·m^−2^·s^−1^ PPFD at a controlled CO_2_ supply (400 mmol CO_2_ mol^−1^ air). Parameters measured included: net photosynthetic rate (Pn), stomatal conductance (gs), intercellular CO_2_ concentration (Ci), and transpiration rate (Tr). These leaf samples were also used for chlorophyll measurement using a mixed extracting solution (acetone:alcohol:distilled water = 4.5:4.5:1) at room temperature for 24 h and then measured with a Bio-Rad SmartSpec Plus spectrophotometer at 663 nm, 645 nm, and 470 nm, respectively, following Tang et al. [88]. Each parameter was determined from 5 biological repeats.

### 4.3. Cell Investigation of odd1 Mutant

We examined the cells of cotyledon epidermis, functional leaf epidermis, and petal in both genotypes with a differential interference contrast (DIC) microscope using an improved method on the basis of Han et al. [89]. The samples were discolored in a mixture containing 84% (*v*/*v*) ethanol and 14% (*v*/*v*) acetic acid at 20 °C for 12 h and subsequently dehydrated through two volume concentrations of ethanol (70% and 100%) three times. After soaking in chloral hydrate (200 g chloral hydrate, 20 g glycerol, 50 mL ddH_2_O) for 30 min, the samples were viewed under a DIC (Imager.Z2, ZEISS, Oberkochen, Germany). Cell area was then calculated using Image J software (v 2.1.4.7, NIH, New York, NY, USA).

### 4.4. Observation of SAM

We took the SAM with hypocotyl or growth points. We used pointed tweezers or surgical blades to peel off the leaves and sundries visible and retained a part of the hypocotyl or stem for easy movement. A stereoscopic microscope (ZEISS V20, Oberkochen, Germany) was used for the observations of SAM.

### 4.5. Pollen Quantity and Vigor Identification

The male flowers were picked on the flowering day. All fresh pollen grains of each flower were stained with Alexander solution, and the number and viability of pollen grains were observed and counted. The pollen quantity per flower was determined by the average of nine selected fields of view. Each parameter was determined from 6 biological repeats. The Alexander storage solution formula (100 mL) was as follows: 20 mL 95% ethanol, 10 mL 1% alcohol-soluble malachite green, 10 g phenol, 10 mL 1% water-soluble acid fuchsin, 1 mL 1% water-soluble orange yellow G, 4 mL glacial acetic acid, 50 mL glycerol, and added water to make up to 100 mL.

### 4.6. Observation of Pollen Germination In Vivo

The pollen of WT and *odd1* were pollinated on the stigma of WT, respectively, and the materials were fixed with FAA fixative for 1 h, rinsed with ddH_2_O 3–4 times, then softened with 8 M NaOH overnight, rinsed with ddH_2_O 3–4 times the next day, then stained with aniline blue for 3 h in the dark. The pollen germination and pollen tube elongation were observed with a fluorescence microscope (Imager.Z2, ZEISS, Oberkochen, Germany).

### 4.7. Gene Preliminary Mapping

The *odd1* mutant was crossed with ‘Chinese long’ 9930 to produce F_2_ for mapping *odd1*. The MutMap method was used for mapping of the *odd1* gene. Thirty mutant plants from the F_2_ population were mixed into a sample pool to extract genomic DNA, construct a sequencing library, and subject it to genome resequencing, and then the genomic resequencing data were compared with the genome sequence of ‘Chinese Long’ 9930. After removing the low-quality SNPs, SNP-index correlation analysis was performed. The closer the SNP-index is to 1, the stronger linkage between the marker and the target gene [90].

### 4.8. RNA Extraction, Library Construction, and Sequencing

Total ribonucleic acid (RNA) of each sample was extracted using the Trizol kit (Ambion^®^, Austin, TX, USA) according to the manufacturer’s instructions. Then, total RNA was purified using RNase-free DNase I (Ambion^®^, Austin, TX, USA). RNA quality was verified using the RNA Nano 6000 Assay Kit of the Bioanalyzer 2100 system (Agilent Technologies, Santa Clara, CA, USA) and was also monitored on 1% RNase-free agarose gel electrophoresis. Next, mRNA was purified from total RNA using poly-T oligo-attached magnetic beads. Fragmentation was carried out using divalent cations under elevated temperature in NEBNext First Strand Synthesis Reaction Buffer (5×). First strand complementary deoxyribonucleic acid (cDNA) was synthesized using random hexamer-primed reverse transcription followed by the synthesis of the second-strand cDNA using RNase H and DNA polymerase I. After adenylation of 3′ ends of DNA fragments, NEB Next Adaptor with hairpin loop structure was ligated to prepare for hybridization. In order to select cDNA fragments of preferentially 250–300 bp in length, the library fragments were purified with an AMPure XP system (Beckman Coulter, Beverly, CA, USA). Adaptor-ligated cDNA fragments were selectively enriched using the NEB Phusion High-Fidelity DNA polymerase, Universal PCR primers, and Index (X) Primer. Products were purified with an AMPure XP system, and library quality was assessed on an Agilent Bioanalyzer 2100 system. The clustering of the index-coded samples was performed on a cBot Cluster Generation System using TruSeq PE Cluster Kit v3-cBot-HS (Illumia) according to the manufacturer’s instructions. The library preparations were sequenced on an Illumina Hiseq platform at Novogene (Tianjin, China), and 125 bp/150 bp paired-end reads were generated.

### 4.9. Bioinformatics Analysis of RNA-Seq Data

Clean data (clean reads) were obtained by removing reads containing adapter, reads containing ploy-N, and low-quality reads from raw data. At the same time, Q20, Q30, and GC content were calculated. All the downstream analyses were based on the clean data with high quality. Clean reads were mapped to the cucumber genome sequence (http://cucurbitgenomics.org/, (accessed on 20 December 2021) v2i) using Hisat2 v2.0.4 (Novogene, Tianjin, China). HTSeq v0.9.1 (Novogene, Tianjin, China) was used to count the read numbers mapped to each gene. Furthermore, the FPKM (fragments per kilobase of transcript sequence per million) of each gene was then calculated based on the length of the gene and reads count mapped to this gene. Genes with low expressions were removed, and only genes with an expression level of at least 1 FRPM in at least two samples were kept for further analysis. Differential expression analysis of two groups was performed using the DESeq R package (1.18.0, Novogene, Tianjin, China). The resulting *p*-values were adjusted using Benjamini and Hochberg’s approach for controlling the false discovery rate. GO enrichment analysis of DEGs was implemented by the GO seq R package. KOBAS software was used to test the statistical enrichment of DEGs in KEGG pathways.

### 4.10. Quantitative Real-Time PCR

To validate the DEGs identified by RNA-seq, we performed qRT-PCR assays using the same samples as those used in transcriptome analysis. The primers used for qRT-PCR are listed in Table S6. qRT-PCR analyses were performed using SYBR Premix Ex Taq (Mei5bio, Beijing, China) with an ABI 7500 Real-Time PCR System (Applied Biosystems, Waltham, MA, USA).

### 4.11. Protein Extraction

The cucumber sample (500 mg per sample) was ground in liquid nitrogen into cell powder and then transferred to a 5 mL centrifuge tube and sonicated by ultrasonic probe for 3 min three times on ice using a high-intensity ultrasonic processor (Scientz, Ningbo, China) in lysis buffer (8 M urea, 1% Triton-100, 10 mM dithiothreitol, and 1% protease inhibitor cocktail (Calbiochem, San Diego, CA, USA)). The remaining debris was removed by centrifugation at 20,000× *g* at 4 °C for 10 min. Finally, the protein was precipitated with cold 200 μL 20% TCA (1/4 volume of the protein solution system) for 2 h at −20 °C. After centrifugation at 12,000× *g* at 4 °C for 10 min, the supernatant was discarded. The remaining precipitate was washed with 1 mL cold acetone three times. The protein was redissolved in 400 μL 8 M urea, and the protein concentration was determined with a BCA kit (Beyotime, Shanghai, China) according to the manufacturer’s instructions. Three biological replicates were performed.

### 4.12. Trypsin Digestion

For digestion, the protein solution was reduced with 5 mM dithiothreitol for 30 min at 56 °C and alkylated with 11 mM iodoacetamide for 15 min at room temperature in darkness. The protein sample was then diluted by adding 100 mM TEAB (tetraethylammonium bromide) to a urea concentration of less than 2 M. Finally, trypsin (Promega, porcine) was added at a 1:50 trypsin-to-protein mass ratio for the first digestion overnight at 37 °C and a 1:100 trypsin-to-protein mass ratio for a second 4 h-digestion at 37 °C.

### 4.13. Tandem Mass Tag Labeling

After trypsin digestion, the peptide was desalted by a Strata X C18 SPE column (Phenomenex) and vacuum-dried. The peptide was reconstituted in 0.5 M TEAB and processed according to the manufacturer’s protocol for TMT kit. Briefly, 1 unit of TMT reagent were thawed and reconstituted in acetonitrile. The peptide mixtures were then incubated for 2 h at room temperature and pooled, desalted, and dried by vacuum centrifugation.

### 4.14. HPLC Fractionation

The tryptic peptides were then fractionated into fractions by high pH reverse-phase HPLC (mobile phase composition: buffer A contains 2% acetonitrile, buffer B contains 98% acetonitrile, pH 9.0; flow rate: 1 mL/min; Agilent Technologies 1260 Infinity) using an Agilent 300 Extend C18 column (5 μm particles, 4.6 mm ID, 250 mm length). Briefly, peptides were first separated with a gradient of 8% to 32% acetonitrile (pH 9.0) over 60 min into 60 fractions. Then, the peptides were combined into 18 fractions and dried by vacuum centrifugation. Each tube had 1 mL of elution buffer. The chromatographic peak of the peptides began from 11 min and ended at 64 min.

### 4.15. LC-MS/MS Analysis

The tryptic peptides were dissolved in 0.1% formic acid (solvent A) and loaded directly onto a home-made reversed-phase analytical column (15-cm length, 75 μmi.d.). The gradient was comprised of an increase from 6% to 23% solvent B (0.1% formic acid in 98% acetonitrile) over 26 min, 23% to 35% in 8 min and climbing to 80% in 3 min, and then holding at 80% for the last 3 min, all at a constant flow rate of 400 nL/min on an EASY-nLC 1000 UPLC (ultra-performance liquid chromatography) system. The peptides were subjected to an NSI (nanospray ionization) source followed by tandem mass spectrometry (MS/MS) in Q Exactive^TM^ Plus (Thermo, Shanghai, China) coupled online to the UPLC. The electrospray voltage applied was 2.0 kV. The *m*/*z* scan range was 350 to 1800 for a full scan, and intact peptides were detected in the Orbitrap at a resolution of 70,000. Peptides were then selected for MS/MS using an NCE setting of 28, and the fragments were detected in the Orbitrap at a resolution of 17,500. A data-dependent procedure that alternated between one MS (mass spectrometry) scan followed by 20 MS/MS scans with 15.0 s dynamic exclusion was conducted. Automatic gain control (AGC) was set at 5E4. Fixed first mass was set as 100 *m*/*z*. The above operations were completed in Jingjie PTM Biolab (Hangzhou, China).

### 4.16. Database Search

The resulting MS/MS data were processed using the Maxquant search engine (v.1.5.2.8, Jingjie PTM Biolab, Hangzhou, China). Tandem mass spectra were searched against a database (http://cucurbitgenomics.org/, accessed on 20 December 2021) made from RNA sequencing of cucumber in this study. Trypsin/P was specified as cleavage enzyme allowing up to 2 missing cleavages. The mass tolerance for precursor ions was set as 20 ppm in the first search and 5 ppm in the main search, and the mass tolerance for fragment ions was set as 0.02 Da. Carbamidomethyl on Cys was specified as fixed modification, and oxidation on Met was specified as variable modifications. FDR (false discovery rate) was adjusted to less than 1%, and minimum score for peptides was set to greater than 40.

### 4.17. Bioinformatic Analysis

Bioinformatic analysis was performed according to previously described protocols [43]. The GO annotation proteome was derived from the UniProt-GOA database (http://www.ebi.ac.uk/GOA/, accessed on 20 December 2021). Proteins were classified by GO annotation into three categories: biological process, cellular compartment, and molecular function. For each category, a two-tailed Fisher’s exact test was employed to test the enrichment of the differentially expressed protein against all identified proteins. The KEGG database was used to annotate the protein pathway [91]. Firstly, the KEGG online service tool KAAS was used to annotated proteins’ KEGG database descriptions. Then the annotation results were mapped on the KEGG pathway database using the KEGG online service tool KEGG mapper. These pathways were classified into hierarchical categories according to the KEGG website. For each category, a two-tailed Fisher’s exact test was used to test the enrichment of the differentially expressed protein against all identified proteins.

## 5. Conclusions

In this study, we characterized a spontaneous mutant *odd1*, which is controlled by a single recessive Mendelian factor that affects almost all the organs of cucumber. Therefore, *odd1* is an ideal material for studying the regulatory mechanisms of growth and development in cucumber. The phenotypic, anatomical, physiological, and cytological analyses demonstrated that the decrease of cell size and cell number, and the shorter and narrower SAM in *odd1* compared with the WT, were the main causes of the size decrease and the abnormal growth and development of organs in *odd1*. Through anatomical observation, pollen vigor identification, cross testing, and observation of pollen germination in vivo, it was found that the main reason for female sterility of *odd1* may be due to the absence of ovules in the mutant. Using the MutMap strategy, the *odd1* gene was successfully located on chromosome 5. Integrated profiling of the transcriptome and proteome further proved that the significantly decreased expression of *CsWOX1*, *CsHP*, and *CsCYCD3*, and the differences in the expression of other genes, hormones, transcription factors, and proteins related to growth and development should be the main reasons for the abnormal growth and development of organs in *odd1*. The model which displays the gene and protein networks in the SAM of *odd1* was presented. Our study not only expanded and deepened the insight into the molecular regulation of organ growth and development in cucumber, but also provided important clues for further studies.

## Figures and Tables

**Table 1:** Photosynthetic parameters of WT and *odd1* mutant at the fruit-setting stage.

Genotype	Chlorophyll a (mg/g FW)	Chlorophyll b (mg/g FW)	Carotenoid (mg/g FW)	Pn (μmol·m^−2^·s^−1^)	Gs (mmol·m^−2^·s^−1^)	Ci (μmol·mol^−1^)	Tr (mmol·m^−2^·s^−1^)
WT	9.34 ± 0.39 a	2.95 ± 0.15 a	1.94 ± 0.09 a	26.51 ± 1.25 a	817.24 ± 109.69 a	242.60 ± 6.27 a	6.11 ± 0.46 a
*odd1*	6.99 ± 0.63 b	2.51 ± 0.23 b	1.52 ± 0.18 b	21.30 ± 1.81 b	465.6 ± 94.81 b	221.00 ± 13.77 b	5.51 ± 0.46 a

**Table 2:** Seed setting by selfing and hybridization.

Types	Seeds
*odd1* (selfing)	no
WT (selfing)	yes
*odd1*♀ × WT♂	no
WT♀ × *odd1*♂	yes

**Table 3:** Segregation of the *odd1* phenotype in the F_1_, F_2_, and F_t_ populations in cucumber.

Populations	# of Plants Observed	# WT	# *odd1*	Expected WT to *odd1* Ratio	*χ*^2^ Value	*p* Value
(Chinese long 9930×*odd1*) F_1_	16	16	0	1:0	/	/
(F_1_ selfing) F_2_	200	143	57	3:1	1.127	0.289
(F_1_×*odd1*) F_t_	200	97	103	1:1	0.125	0.724

**Table 4:** Summary of the number of proteins and mRNA detected in WT and *odd1* mutant.

Category	Proteins	mRNAs
Unique protein/gene detected	6283	24,118
Significantly changed proteins/genes	356	565
Up-regulated	176	314
Down-regulated	180	251

**Table 5:** List of the 42 cor-DEG-DEP genes that were regulated at both transcriptional and translational levels.

Gene ID	Gene Description	Fold Change (*odd1*/WT)	*p* Value	Regulated Type
*Csa2G238880*	Non-symbiotic hemoglobin 1	0.271	0.000162	Down
*Csa3G872170*	Gibberellin-regulated protein	2.382	0.000364	Up
*Csa6G085120*	Hfr-2-like protein	3.154	0.000364	Up
*Csa1G187170*	Unknown protein	2.141	0.000456	Up
*Csa2G055560*	Choline dehydrogenase	2.113	0.000776	Up
*Csa6G085110*	Hfr-2-like protein	3.462	0.000864	Up
*Csa6G109750*	UDP-glucosyltransferase, putative	1.423	0.000962	Up
*Csa2G023940*	Lipoxygenase	2.198	0.000978	Up
*Csa7G073410*	Leucine-rich repeat receptor-likeserine/threonine-protein kinase	1.588	0.000979	Up
*Csa5G410730*	Glutamine synthetase	1.875	0.00112	Up
*Csa1G188680*	Xyloglucan endotransglucosylase/hydrolase	1.56	0.00136	Up
*Csa3G903550*	Putative cytochrome P450superfamily protein	1.742	0.00164	Up
*Csa2G009470*	Betaine aldehyde dehydrogenase	0.778	0.00184	Down
*Csa1G151000*	Stress responsive A/B barrel domainfamily protein	1.386	0.00268	Up
*Csa6G088160*	Cytochrome P450, putative	1.686	0.00274	Up
*Csa6G522760*	Ycf23 protein	0.701	0.00274	Down
*Csa3G698490*	Cytochrome P450	1.346	0.00338	Up
*Csa7G414480*	Short-chain dehydrogenase/reductasefamily protein	1.362	0.00356	Up
*Csa5G224130*	Cytochrome P450	1.307	0.0037	Up
*Csa1G541390*	Phosphatidylinositol transfer protein sfh5	0.72	0.00418	Down
*Csa7G447020*	Probable peptide/nitrate transporter	1.458	0.00504	Up
*Csa3G903540*	Putative cytochrome P450superfamily protein	1.795	0.00516	Up
*Csa6G490110*	Methylesterase	1.585	0.0062	Up
*Csa3G778270*	Short-chain dehydrogenase/reductase 2	1.308	0.00648	Up
*Csa3G778280*	Short-chain dehydrogenase/reductase 2	1.44	0.00694	Up
*Csa6G088710*	Cytochrome P450	1.547	0.00728	Up
*Csa6G088700*	Anthranilate N-benzoyltransferaseprotein, putative	1.647	0.00966	Up
*Csa7G451920*	Putative phosphatase	0.526	0.00994	Down
*Csa1G044890*	Cytochrome P450	1.907	0.014	Up
*Csa4G285730*	Peroxidase	1.668	0.0154	Up
*Csa6G183190*	Asparagine synthetase	1.52	0.0162	Up
*Csa1G611290*	Beta-glucosidase D7	1.566	0.0171	Up
*Csa4G285740*	Peroxidase	1.663	0.0172	Up
*Csa3G556210*	Glycerophosphodiesterphosphodiesterase	0.51	0.0204	Down
*Csa4G288610*	Lipoxygenase	1.425	0.0216	Up
*Csa1G044880*	Short-chain dehydrogenase/reductase 1	1.363	0.0232	Up
*Csa4G285760*	Peroxidase	1.352	0.0237	Up
*Csa6G088170*	Cytochrome P450	1.304	0.0253	Up
*Csa5G576590*	Auxin efflux carrier	1.279	0.0255	Up
*Csa5G636450*	Lipid A export ATP-binding/permeaseprotein MsbA	1.269	0.0269	Up
*Csa3G172370*	MLP	1.333	0.035	Up
*Csa3G435030*	Profilin	1.401	0.0496	Up

## Data Availability

The data presented in this study are available in this article and the Supplementary Materials.

## Supplementary Materials

The following supporting information can be downloaded at: https://www.mdpi.com/article/10.3390/ijms23105843/s1. Figure S1. Pearson correlation analysis of transcriptomic and proteomic data between all samples, respectively. Figure S2. Principal component analysis of transcriptomic and proteomic data between all samples, respectively. Figure S3. qRT-PCR validation of DEGs identified by RNA-seq. Figure S4. Transcriptomics and proteomics identified compared venn diagram. Table S1. The quality of re-sequencing data. Table S2. Summary of transcriptome sequencing data. Table S3. Mapped regions distribution in the reference genome. Table S4. List of transcription factors that were down-regulated in *odd1* mutant. Table S5. List of transcription factors that were up-regulated in *odd1* mutant. Table S6. Primers for qRT-PCR.
