# Supplementary material for: Morphological Characterization and Integrated Transcriptome and Proteome Analysis of Organ Development Defective 1 (odd1) Mutant in Cucumis sativus L
Source: Int J Mol Sci. 2022 May 23;23(10):5843. doi: 10.3390/ijms23105843 (PMC9145247; doi:10.3390/ijms23105843)
Supplement: Supplementary file 1 [file ijms-23-05843-s001.zip › ijms-1715341-supplementary.pdf]

Supplementary figures and tables

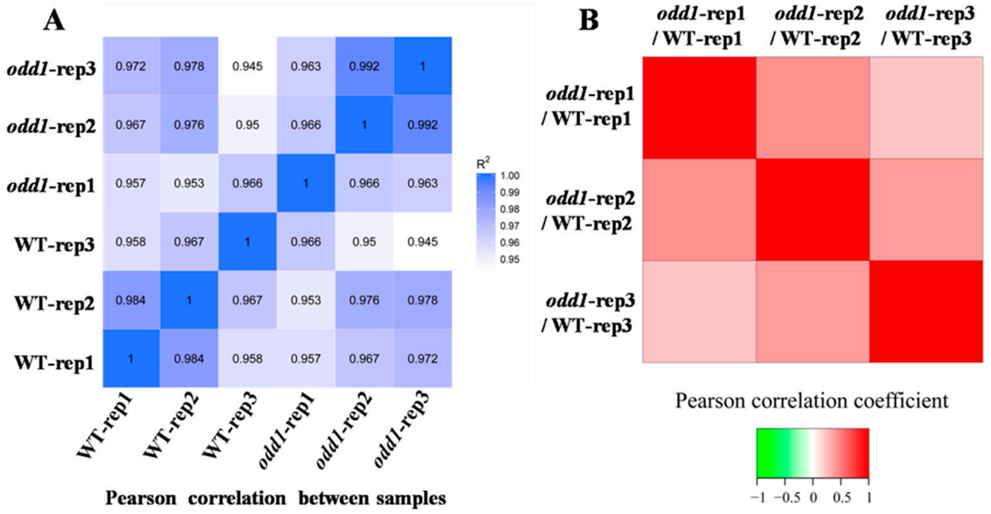

Figure S1. Pearson correlation analysis of transcriptomic and proteomic data between all samples, respectively. (**A**) Pearson correlation analysis of transcriptomic data. (**B**) Pearson correlation analysis of proteomic data. The color code indicates Pearson correlation coefficient value and the color scale of the heat map ranges from saturated green (value, −1) to saturated red (value, 1).

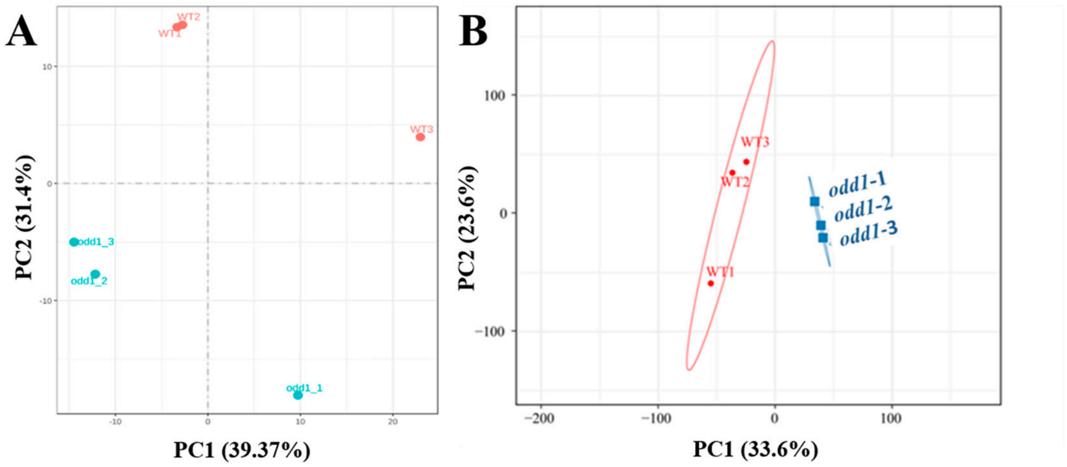

Figure S2. Principal component analysis of transcriptomic and proteomic data between all samples, respectively. (**A**) Principal component analysis at the transcriptome level. (**B**) Principal component analysis at the proteome level.

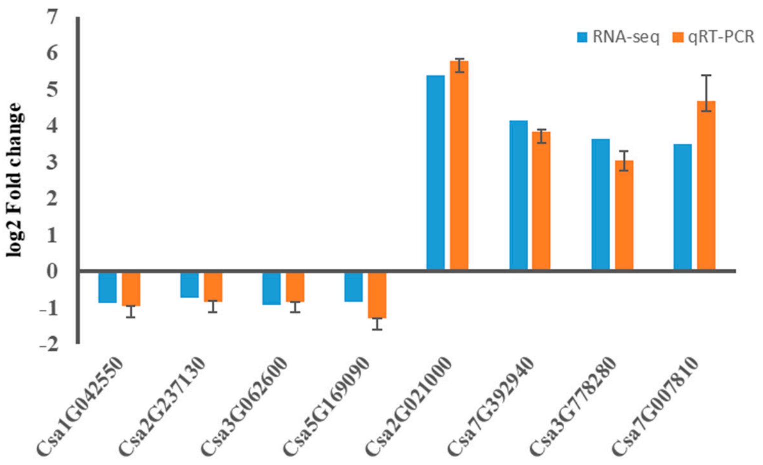

Figure S3. qRT-PCR validation of DEGs identified by RNA-seq.

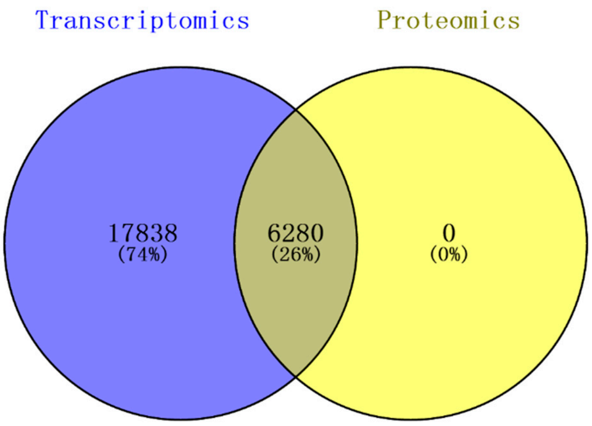

Figure S4. Transcriptomics and proteomics identified compared venn diagram.

Table S1. The quality of re-sequencing data.

Sample	Raw reads	Clean bases	Mapped (%)	Q20 (%)	Q30 (%)	GC content (%)
odd1	48664295	45992885	92.4	95.77	90.79	37.65

Table S2. Summary of transcriptome sequencing data.

Sample	Raw reads	Clean reads (Clean/All)	Mapped Reads (Mapped/Clean)	Uniquely mapped (Unique/Clean)	Q20 (%)	GC content (%)
WT-rep1	56857254	54862040(96.49%)	52588770 (95.86%)	51649382 (94.14%)	97.23	42.93
WT-rep2	63656560	62760600(98.59%)	60223314 (95.96%)	59122631 (94.20%)	97.11	43.16
WT-rep3	52118766	51169978(98.18%)	49175600 (96.10%)	48348213 (94.49%)	98.08	42.08
odd1-rep1	42777848	41132430(96.15%)	39215014 (95.34%)	38549174 (93.72%)	97.85	42.00
odd1-rep2	51930126	51164624(98.53%)	49059426 (95.89%)	48200934 (94.21%)	97.17	42.99
odd1-rep3	55159510	54155680(98.18%)	52063995 (96.14%)	51135218 (94.42%)	97.31	43.18

Table S3. Mapped regions distribution in the reference genome.

Sample	Intergenic (%)	Exon (%)	Intron (%)
WT-rep1	5.8	92.1	2.1
WT-rep2	6.0	92.0	2.0
WT-rep3	6.9	90.7	2.4
odd1-rep1	6.6	91.5	1.9
odd1-rep2	5.9	91.8	2.3
odd1-rep3	5.8	92.0	2.2

Table S4. List of transcription factors that were down-regulated in *odd1* mutant.

Gene ID	Gene description	$\vert log_2$ (fold change)$\vert$	Padj (adjusted P-value)
**AP2-EREBP**			
Csa5G155570	Dehydration responsive element binding transcription factor 1B	5.5528	0.0051271
Csa1G075060	Ethylene-responsive transcription factor, putative	2.2624	0.040152
Csa4G630010	Ethylene-responsive transcription factor 6	1.8708	0.045959
Csa7G447150	Ethylene-responsive transcription factor 6	1.5621	0.0027254
Csa5G612310	AP2-like ethylene-responsive transcription factor	1.4571	0.01555
Csa3G357110	Ethylene responsive transcription factor 1a	0.90386	0.008539
**C2H2**			
Csa3G141860	Zinc finger C2H2-type contained transcriptional regulator superman	3.1593	0.0069897
Csa7G071440	Zinc finger C2H2-like contained protein	1.6484	0.044601
Csa7G428260	Zinc finger C2H2-type contained protein	1.4741	0.00018434
**HSF**			
Csa6G169310	Heat stress transcription factor A-4a	2.3722	0.0015535
Csa6G490870	Heat stress transcription factor	1.3613	0.0033273
**HB**			
Csa1G042780	WUSCHEL-related homeobox 1	2.2545	0.0000263
Csa4G043850	Homeobox-leucine zipper protein	1.1622	0.0023245
**WRKY**			
Csa3G730800	WRKY transcription factor 17	1.9597	0.034386
Csa4G051470	WRKY transcription factor 21	1.8208	0.0056665
Csa2G297180	WRKY transcription factor 16	1.4414	0.00049001
Csa3G119700	Putative WRKY transcription factor 30	1.1134	0.013385
**bHLH**			
Csa3G354520	bHLH domain-containing transcription factor	1.8743	0.004005
**GRAS**			
Csa6G495010	GRAS family transcription factor	1.7225	0.0017211
Csa3G405510	Scarecrow-like 1 transcription factor	1.3704	0.004545
**MYB**			

Csa3G826690	MYB family transcription factor-like protein	0.97522	0.0046356
Csa1G033200	MYB transcription factor	0.74405	0.036368
**Others**			
Csa2G295940	LOB domain-containing protein	1.6026	0.00000114
Csa1G051590	Squamosa promoter binding protein	1.1462	0.0045259
Csa7G448770	Zinc finger CCCH domain-containing protein	1.0682	0.0063682
Csa7G290470	FHA domain containing protein, ex-pressed	1.0374	0.004545
Csa2G223670	Protein tesmin/TSO1-like CXC 2	0.99262	0.011514
Csa3G645940	Protein TIFY	0.79419	0.035278
Csa3G113370	NAC domain protein	0.79032	0.014446
Csa6G445000	DNA-binding protein S1FA, putative	0.75534	0.029907
Csa4G051430	High mobility group family, contains high mobility group (HMG) box do-main	0.72699	0.025411

Table S5. List of transcription factors that were up-regulated in *odd1* mutant.

Gene ID	Gene description	$\vert log_2$ (fold change)$\vert$	padj(adjusted P-value)
**bHLH**			
Csa4G312280	bHLH domain-containing transcription factor	3.0384	0.001786
Csa6G011720	Transcription factor bHLH51	1.2575	0.00014729
Csa1G042340	bHLH domain-containing transcription factor	1.1364	0.00079987
Csa7G428890	Transcription factor bHLH122	0.87739	0.024503
**bZIP**			
Csa2G416070	bZIP transcription factor family protein	2.4188	0.0257
Csa5G642710	bZIP transcription factor	1.1657	0.024835
**HB**			
Csa5G635430	Homeobox-leucine zipper-like protein	2.0763	0.00000000852
Csa4G645830	Knotted-like homeobox protein	1.5393	0.000000742
Csa6G501990	Homeobox-leucine zipper protein	0.73265	0.037691
**MYB**			
Csa1G042350	MYB transcription factor	1.8445	0.000000219
Csa7G413890	MYB-like transcription factor MYB 5	1.2809	0.007506
Csa6G040640	Putative MYB transcription factor	1.1889	0.00098148
Csa2G100550	MYB transcription factor	1.0642	0.020392
**ARF**			
Csa6G291920	Auxin response factor 5	1.7374	0.000000000000229
Csa6G518210	Auxin response factor, putative	1.6822	0.00000000000413
Csa6G524670	Auxin response factor 3	0.65105	0.045938
**WRKY**			
Csa5G223070	WRKY transcription factor, putative	1.6167	0.002022
Csa1G547510	WRKY transcription factor 14-1	1.5325	0.037691
**NAC**			
Csa4G629480	NAC domain protein	1.4533	0.00013625
Csa1G038340	NAC domain-containing protein 21/22, putative	0.87849	0.0062603
Csa3G824990	NAC domain protein	0.81129	0.018391

**C2H2**			
Csa1G043020	Putative zinc finger C2H2-type contained protein	1.343	0.00000877
Csa5G092930	Putative zinc finger C2H2-type contained protein	1.1256	0.00079907
**LOB**			
Csa3G278920	LOB domain-containing protein	1.2343	0.0000894
Csa6G012240	LOB domain-containing protein, putative	0.998	0.0013866
**TCP**			
Csa1G020890	TCP, transcription factor CYCLOIDEA	1.1351	0.013385
Csa1G033030	TCP transcription factor,putative	0.82854	0.024668
**AUX/IAA**			
Csa7G378520	Auxin-responsive protein	1.129	0.0032235
Csa1G397130	Auxin-responsive protein	1.032	0.001255
Csa3G143570	Auxin-responsive protein	0.72205	0.020911
**Others**			
Csa6G040600	GRAS, putative nodulation signaling pathway 2 protein	1.4767	0.00049001
Csa3G904140	Two-component response regulator-like protein	1.2454	0.0062603
Csa1G043000	G2-like	1.0396	0.0057619
Csa6G039540	C2C2-CO-like, CONSTANS-like zinc finger protein	0.94739	0.035103
Csa6G432270	SRS, short internode related sequence	0.88733	0.0066296
Csa3G751470	GRF, putative growth-regulating factor 6	0.84733	0.029301
Csa6G091830	AP2-EREBP, ethylene-responsive transcription factor	0.71479	0.041473

Table S6. Primers for qRT-PCR.

Primer name	Sequence (5′-3′)
Csa1G042550-F	GCCAGGCTAGAAGCAGAAC
Csa1G042550-R	TGCTGGTCCTCCTCAAGTTT
Csa3G062600-F	GAGACACTGCTGAAGCTG
Csa3G062600-R	CAGTTTGCTCTCTGCAAGGT
Csa2G237130-F	GAGATTGATAGAAAGCAAGCTG
Csa2G237130-R	TGGCATGGTGGTGAATACG
Csa5G169090-F	AGTGACCGGCAGATGGT
Csa5G169090-R	GATGCCAAAGCTCCAACA
Csa2G021000-F	CGATCAGTGCCCATTTTACGT
Csa2G021000-R	CGTGTTTCAAGCGTTCACCA
Csa7G392940-F	TGACATCGCCACTCGATTCC
Csa7G392940-R	GGAATCGAGTGGCGATGTCA
Csa7G007810-F	CCGCAAATACTCAACGTT
Csa7G007810-R	TTTGATGAGATCCATATC
Csa3G778280-F	CAACTACTTGGCAAAGGAG
Csa3G778280-R	GTTTGATGCCGAGGAAGACA
